# Efficient elimination of MELAS-associated m.3243G mutant mitochondrial DNA by an engineered mitoARCUS nuclease

**DOI:** 10.1038/s42255-023-00932-6

**Published:** 2023-11-30

**Authors:** Wendy K. Shoop, Janel Lape, Megan Trum, Alea Powell, Emma Sevigny, Adam Mischler, Sandra R. Bacman, Flavia Fontanesi, Jeff Smith, Derek Jantz, Cassandra L. Gorsuch, Carlos T. Moraes

**Affiliations:** 1 Precision BioSciences, Durham, NC USA; 2 Department of Biochemistry and Molecular Biology, University of Miami Miller School of Medicine, Miami, FL USA; 3 Department of Neurology, University of Miami Miller School of Medicine, Miami, FL USA

**Keywords:** Molecular medicine, Medical genomics, Metabolism, Mitochondria

## Abstract

Nuclease-mediated editing of heteroplasmic mitochondrial DNA (mtDNA) seeks to preferentially cleave and eliminate mutant mtDNA, leaving wild-type genomes to repopulate the cell and shift mtDNA heteroplasmy. Various technologies are available, but many suffer from limitations based on size and/or specificity. The use of ARCUS nucleases, derived from naturally occurring I-*Cre*I, avoids these pitfalls due to their small size, single-component protein structure and high specificity resulting from a robust protein-engineering process. Here we describe the development of a mitochondrial-targeted ARCUS (mitoARCUS) nuclease designed to target one of the most common pathogenic mtDNA mutations, m.3243A>G. mitoARCUS robustly eliminated mutant mtDNA without cutting wild-type mtDNA, allowing for shifts in heteroplasmy and concomitant improvements in mitochondrial protein steady-state levels and respiration. In vivo efficacy was demonstrated using a m.3243A>G xenograft mouse model with mitoARCUS delivered systemically by adeno-associated virus. Together, these data support the development of mitoARCUS as an in vivo gene-editing therapeutic for m.3243A>G-associated diseases.

## Abstract (web summary)

Shoop et al. develop mitoARCUS, a mitochondria-targeted nuclease with high specificity, to correct a relatively common pathogenic mtDNA mutation, allowing for beneficial shifts in heteroplasmy while reducing nuclear off-target gene editing.

## Main

Mutations that impair genes involved in mitochondrial function are responsible for a heterogeneous group of disorders characterized by defective mitochondrial ATP production^1^. Mitochondrial diseases can be due to mutations in genes necessary for mitochondrial function that are expressed from either nuclear or mitochondrial genomes^2^. Mitochondrial DNA encodes for 13 subunits of the oxidative phosphorylation (OXPHOS) system, as well as two ribosomal RNAs and 22 transfer RNAs that are necessary to support mitochondrial translation^3,4^. Mitochondrial diseases affect approximately one in 5,000 people and, collectively, are one of the most common hereditary metabolic disorders^5,6^.

Mitochondrial encephalomyopathy, lactic acidosis and stroke-like episodes (MELAS) comprise a multisystemic mitochondrial disease that was first identified as a clinical entity in 1984 (ref. ^7^). While data on disease incidence are limited, a prospective cohort study in Japan estimated prevalence to be one per 500,000 individuals^8^. Symptoms can vary between individuals but typically include recurrent stroke-like episodes, encephalopathy and the accumulation of lactic acid in the bloodstream^9^. The median survival time for fully symptomatic patients is approximately 17 years following the onset of neurologic symptoms, with an average observed age at death of 34.5 ± 19 years^10^. Treatment is palliative in nature and there are currently no cures available for this disease. Mutations in multiple mtDNA genes have been implicated in MELAS, including *MT-TL1*, *MT-TK*, *MT-T*Q and *MT-ND5* among others^11,12^. The most prevalent mtDNA mutations exist in *MT-TL1*, which encodes mitochondrial tRNA^Leu(UUR)^. Specifically, a point mutation at mtDNA position 3243 (m.3243A>G) is responsible for >80% of MELAS cases^11^. In addition to MELAS, this mutation has been associated with diverse clinical phenotypes including hearing loss, migraine, muscle weakness and diabetes^13–15^. Overall, the population prevalence of the m.3243A>G mutation is estimated to be as high as one in 400 individuals^16^.

Unlike the diploid nuclear genome mtDNA is polyploid, with hundreds to thousands of copies present per mammalian cell^17^. The multicopy nature of the genome leads to the coexistence of mutant and wild-type genomes in the same cell, a state known as heteroplasmy^18–20^. The presence of wild-type genomes offsets the impact of the mutant genomes until the percentage of mutant mtDNA exceeds a particular threshold, at which point clinical symptoms manifest^21–23^. Therefore, it is not only the exact mutation that can impact disease presentation and progression, but also the mutation load and tissues affected, which can complicate diagnosis and disease management^2^.

In recent years gene-editing technologies have been adapted to edit these heteroplasmic mtDNA mutations^24–27^. Because there is no efficient double-strand break (DSB) repair mechanism in mitochondria, any mtDNA molecules that are linearized by a nuclease will be rapidly degraded^28–31^. Following this depletion of mtDNA copies there is a tightly controlled, yet incompletely understood, mechanism for maintaining mtDNA copy number that results in the replication of any remaining genomes^32–34^. Thus, specific cleavage of mutant mtDNA genomes leads to their degradation and then subsequent repopulation with residual (mostly wild-type) mtDNA. This therapeutic approach to shifting mtDNA heteroplasmy below the disease threshold has proven to be a powerful and efficient way to eliminate mutant mtDNA in both cultured cells and animal models^35–42^. However, the specificity of the nuclease used to target the mutant haplotype is a concern in regard to any of these heteroplasmic point mutations because only a single nucleotide differs between mutant and wild-type sequences and indiscriminate mtDNA cleavage could be catastrophic.

ARCUS nucleases are re-engineered from I-*Cre*I, an endonuclease that recognizes and cleaves a 22-base-pair (bp) DNA sequence found in the chloroplast genome of *Chlamydomonas reinhardtii*^43^. Different ARCUS nucleases have been used to generate allogeneic CAR T cells for the treatment of certain blood cancers, knockout genes involved in cholesterol metabolism and transthyretin amyloid fibril production, as well as to inactivate hepatitis B virus^44–48^. We recently showed that a mitochondrial-targeted ARCUS (mitoARCUS) nuclease could shift heteroplasmy in m.5024 C>T cultured cells and heteroplasmic mice following systemic delivery by adeno-associated virus 9 (AAV9)^42^. ARCUS has several advantages over other programmable gene-editing technologies for the purposes of mitochondrial gene editing. Unlike the large, dimeric zinc-finger nucleases (ZFNs) and transcription activator-like effector nucleases (TALENs), ARCUS is a single-component protein that permits both DNA recognition and DSB generation^47^. Its single-protein attribute allows for simplified viral delivery because only one AAV vector is needed to fit the ~1,100 bp coding sequence^47^. Finally, the amino acids involved in substrate recognition can be optimized to improve both activity and specificity, leading to highly specific nucleases^45^. Based on these features and the prevalence of the m.3243G mutation, we developed and optimized a mitoARCUS enzyme for specific cleavage of mutant m.3243G mtDNA. We demonstrated a high degree of specificity of the candidate enzyme and showed that shifts in heteroplasmy induced by this nuclease resulted in improvements in mitochondrial function, without any toxicity associated with the transient mtDNA depletion that was induced by the elimination of mutant genomes. Additionally, we characterized nuclear off-target editing of the candidate mitoARCUS nuclease and determined that nuclear editing could be further safeguarded by inclusion in the construct of a nuclear export signal (NES), in addition to a mitochondrial targeting sequence (MTS). Finally we developed a novel mouse model and demonstrated proof of concept for this therapeutic approach in vivo with nuclease delivery by systemic AAV administration.

## Results

### mitoARCUS localizes to the mitochondrial matrix

**Fig. 1:**
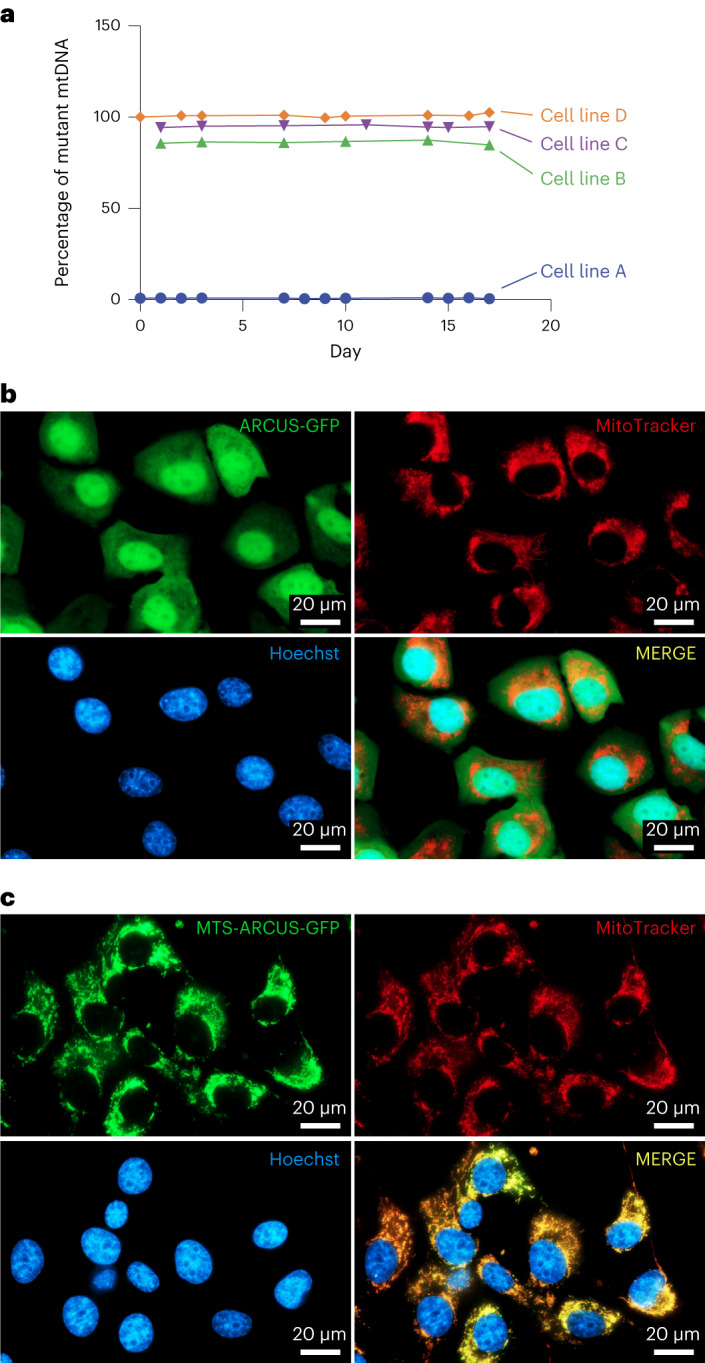
Addition of an MTS to ARCUS (mitoARCUS) localizes the nuclease to mitochondria. **a**–**c**, Various m.3243A>G cybrid cell lines (cell lines A–D) were used for subsequent experiments. Cell line C (95% mutant) was used to evaluate ARCUS subcellular localization 24 h following mRNA nucleofection of ARCUS, either without a targeting sequence or with an MTS fused at the N terminus. m.3243A>G heteroplasmy analysis of various cybrid cell lines over 17 days in culture (**a**). Fluorescence microscopy of ARCUS lacking a targeting sequence (**b**). The experiment was performed once. Fluorescence microscopy of ARCUS with an MTS at the N terminus (mitoARCUS) (**c**). The experiment was performed once. ARCUS is shown in green, mitochondria (stained with MitoTracker) in red and nuclei (stained with Hoechst 33342) in blue; colocalization of ARCUS and MitoTracker is depicted in yellow (**b**,**c**).

To evaluate the ability of mitoARCUS to specifically cleave m.3243G mutant mtDNA, cell lines containing varying levels of the mutation were generated. All of these cell lines were isolated from the same parental m.3243A>G cybrid cell line. Levels of the m.3243G mutation of each untreated line were maintained throughout the duration of the experiments (Fig. 1a).

Many nuclear-encoded proteins that localize to mitochondria do so through a naturally occurring MTS on the peptide. Therefore, exogenous proteins can be similarly trafficked to the mitochondrial matrix by inclusion of an MTS. We chose to use a combination of COX8A and SU9 MTS in tandem, because this combination has previously been shown to result in efficient mitochondrial localization^39,42^. To determine whether the addition of this MTS combination to ARCUS would effectively result in mitochondrial trafficking in MELAS cybrid cells, cell line C (95% mutant) was used to evaluate ARCUS subcellular localization following nucleofection. The ARCUS protein, devoid of a specific targeting sequence, appeared diffuse throughout the cytoplasm and nucleus (Fig. 1b) while the addition of the N-terminal MTS permitted efficient mitochondrial localization, as demonstrated by the overlay with MitoTracker (Fig. 1c). Unless otherwise noted, this MTS was used in all subsequent experiments to understand the impact of mtDNA targeting.

### Engineering a nuclease that specifically eliminates m.3243G mutant mtDNA

**Fig. 2:**
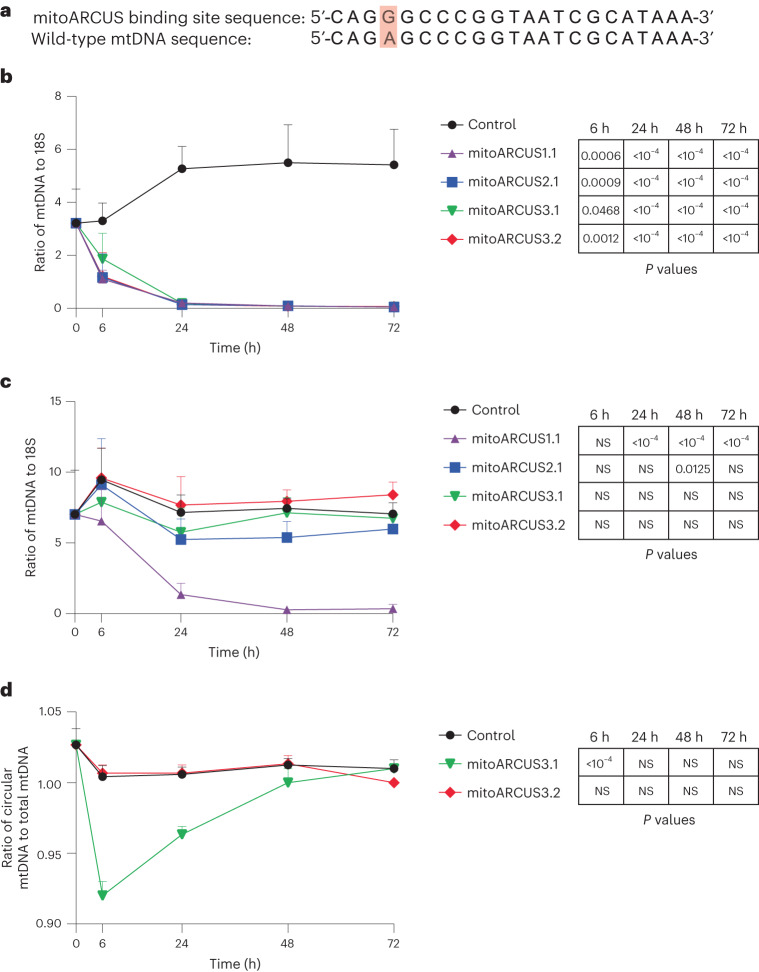
mitoARCUS3.2 efficiently eliminates m.3243G mutant mtDNA without cutting m.3243A wild-type mtDNA. **a**–**d**, Various mitoARCUS enzymes were designed to bind and cleave mutant m.3243G; however, wild-type m.3243A differs by only a single nucleotide and thus enzyme specificity is imperative. Enzymes were evaluated in cells containing either 100 or 0% mutant (100% wild-type) mtDNA for mtDNA cleavage and loss over time. All mRNAs, including the MTS-GFP control. were dosed at 1.5 × 10^5^ mRNA copies per cell. The 22 bp (5' > 3') target site for m.3243G-targeting mitoARCUS and the corresponding wild-type mtDNA sequence (**a**). Position m.3243 is highlighted in red. mtDNA copy number over time in cell line D (100% mutant) (**b**). mtDNA copy number over time in cell line A (0% mutant) (**c**). mtDNA linearization at position m.3243 in cell line A (0% mutant) (**d**). A ratio <1.0 indicates that cleavage has occurred at wild-type m.3243 A. Data encompass three independent experiments and are shown as mean + s.d. Statistical analysis was performed using one-way ANOVA. NS at *P* > 0.05; all other *P* values indicated. 18S, 18S rDNA.

To test a gene-editing approach for shifting m.3243A>G heteroplasmy, multiple mitoARCUS nucleases containing slightly different amino acid sequences were designed to target the mutant m.3243G mtDNA sequence. While achieving on-target cleavage at the mutant mtDNA sequence was critical, it was imperative that a nuclease could discriminate precisely between mutant and wild-type mtDNA sequences, which differ by only a single nucleotide (Fig. 2a). Therefore, the primary focus during nuclease generation and optimization was identification of a nuclease that cleaved the mutant sequence but not the wild-type sequence.

A homoplasmic mutant cell line (cell line D, 100% mutant) and a homoplasmic wild-type cell line (cell line A, 0% mutant) were used to evaluate the various nucleases. Because these particular cell lines did not contain heteroplasmic mtDNA, the outcome of a nuclease-induced DSB would be depletion of the linearized mtDNA molecule. Therefore, these two cell lines were used as tools to evaluate nuclease activity, observed as mtDNA depletion in the 100% mutant cell line, and nuclease specificity, observed as a lack of mtDNA depletion in the 0% mutant cell line. Messenger RNAs encoding each of the various nucleases or an MTS-green fluorescent protein (GFP) control were nucleofected into both cell lines, and cellular DNA was collected and evaluated for mtDNA copy number along a time course.

MitoARCUS1.1 was found to robustly eliminate mutant mtDNA, leading to a significant reduction in mtDNA copy number compared with control at all time points (Fig. 2b). By 72 h there were 5.41 ± 1.35 copies of mtDNA per copy of nuclear 18S ribosomal DNA present in the control whereas mitoARCUS1.1-treated cells had just 0.05 ± 0.05 copies of mtDNA per copy of 18S rDNA. However, mitoARCUS1.1 also cleaved the wild-type sequence, resulting in a marked reduction in wild-type mtDNA beginning at 24 h that was sustained at 72 h (Fig. 2c). Because there were 7.05 ± 0.80 and 0.34 ± 0.32 copies of mtDNA per copy of 18S rDNA at 72 h in control and mitoARCUS1.1-treated cells, respectively, optimization was required to improve the specificity of the enzyme for the m.3243 nucleotide.

We completed a round of nuclease optimization with a focus on improving specificity against the wild-type mtDNA sequence while still maintaining on-target activity. This generated mitoARCUS2.1, which was evaluated as described above. Again mitoARCUS2.1 demonstrated potent reductions in mutant mtDNA (Fig. 2b), resulting in just 0.05 ± 0.04 copies of mtDNA per copy of 18S rDNA remaining at 72 h, indicating that on-target activity had not been sacrificed during the optimization process. While wild-type mtDNA copy number at 72 h was not significantly different from control (7.05 ± 0.80 and 5.98 ± 1.15 copies of mtDNA per copy of 18S rDNA in control and mitoARCUS2.1-treated cells, respectively) and thus demonstrated an improvement in specificity over mitoARCUS1.1, there was a transient but significant depletion of wild-type mtDNA observed at 48 h (Fig. 2c). At this time point there were 7.45 ± 0.69 and 5.38 ± 1.13 copies of mtDNA per copy of 18S rDNA in control and mitoARCUS2.1-treated cells, respectively. Therefore a third round of nuclease optimization was completed, again with a focus on prevention of wild-type mtDNA cleavage.

MitoARCUS3.1 and mitoARCUS3.2 were evaluated in the same manner. Once again, both enzymes resulted in a near-complete elimination of mutant mtDNA by 72 h, with just 0.05 ± 0.03 and 0.07 ± 0.02 copies of mtDNA present per copy of 18S rDNA, respectively (Fig. 2b). However, unlike the earlier-generation nucleases, mitoARCUS3.1 and mitoARCUS3.2 did not generate a statistically significant depletion of wild-type mtDNA compared with control at any of the time points, indicating a high degree of specificity of both enzymes for the mutant mtDNA sequence (Fig. 2c).

To differentiate these two nucleases in terms of specificity against the wild-type mtDNA sequence, linearization of mtDNA at position m.3243 was evaluated. We reasoned that a very minimal amount of mitoARCUS-induced DSBs might not translate to appreciable levels of mtDNA depletion in the context of regular mtDNA replication and degradation. However, the presence of linearized mtDNA molecules at m.3243 following nuclease-induced DSB and before their degradation by endogenous mitochondrial exonucleases could represent a more sensitive metric for evaluation of nuclease activity at the wild-type mtDNA sequence^28,31^. Therefore we analysed the same cellular DNA samples from cell line A (0% mutant) and reported mtDNA linearization as the ratio of mtDNA molecules that are circular at position m.3243 out of the total number of mtDNA molecules (Fig. 2d). At 6 h the ratio of circular to total mtDNA at position m.3243 was 1.00 ± 0.01, 0.92 ± 0.01 and 1.01 ± 0.01 for control, mitoARCUS3.1- and mitoARCUS3.2-treated cells, respectively. A ratio of 1.00 indicated that all mtDNA genomes present were circular, whereas a ratio of 0.92 indicated that 92% of mtDNA genomes present were circular and 8% were linear at m.3243. Hence, mitoARCUS3.1 caused a significant decrease in the percentage of circular mtDNA at m.3243 while mitoARCUS3.2 was statistically insignificant from control across all time points. Even with twice the amount of mitoARCUS mRNA and with additional early time points, we did not observe a significant reduction (Supplementary Fig. 1a) or linearization (Supplementary Fig. 1b) of wild-type mtDNA genomes with mitoARCUS3.2. Based on its high level of specificity, mitoARCUS3.2 was used for subsequent experiments.

### mitoARCUS shifts heteroplasmy and improves mitochondrial function

Once a highly specific nuclease had been identified we sought to understand its impact in the context of a heteroplasmic cell line, specifically cell line C (95% mutant). This cell line was selected due to its significant respiratory impairment (Supplementary Fig. 2a–c) compared with that of wild-type cells (cell line A). mRNA encoding either mitoARCUS3.2 or MTS-GFP was nucleofected into cell line C, with mitoARCUS3.2 mRNA dosed at tenfold dilutions starting at 1 × 10^5^ mRNA copies per cell. At days 3 and 7 post transfection, cellular DNA was collected for heteroplasmy and mtDNA copy number analysis, lysates were collected for protein analysis and live cells were analysed for respiration.

**Fig. 3:**
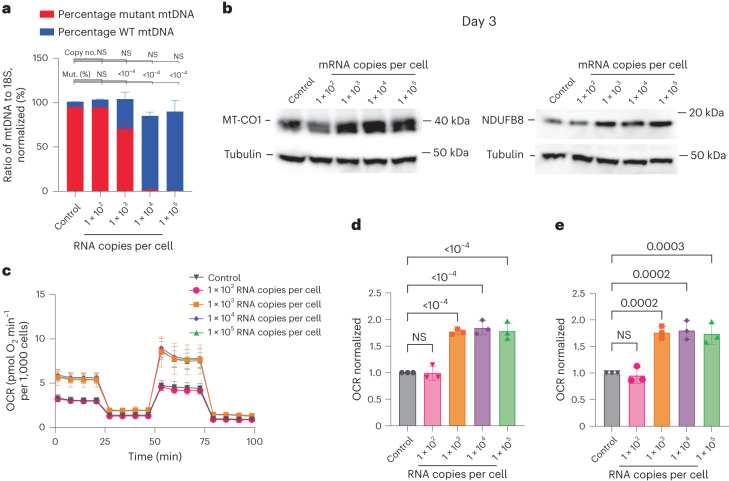
mitoARCUS3.2 shifts heteroplasmy in 95% of m.3243G mutant cybrid cells, resulting in increased steady-state levels of mitochondrial proteins and increased respiration at day 3. **a**–**e**, Cell line C (95% mutant) was nucleofected with either an MTS-GFP control or mitoARCUS3.2 mRNA at tenfold dilutions, starting at 1 × 10^5^ mRNA copies per cell. Cellular DNA was collected at day 3 for mtDNA heteroplasmy and mtDNA copy number analysis (**a**), protein lysates were collected for immunoblot (**b**) and live cells were analysed for respiration (**c**–**e**). mtDNA heteroplasmy, normalized to mtDNA copy number of control (**a**). Immunoblots showing MT-CO1, NDUFB8 and alpha-tubulin steady-state levels (**b**). Seahorse Cell Mito Stress Test (**c**). Basal respiration, normalized to control (**d**). Maximal respiration, normalized to control (**e**). Data encompass three independent experiments and are shown as mean ± s.d. Statistical analysis was performed using one-way ANOVA on raw data. NS, *P* > 0.05; all other *P* values indicated. Source data

At day 3 there were no significant changes observed in mtDNA copy number across any of the mitoARCUS-treated conditions (Fig. 3a). However, cells treated with 1 × 10^3^, 1 × 10^4^ and 1 × 10^5^ mRNA copies per cell exhibited significant reductions in the percentage of mutant mtDNA present. These cells contained 68.7 ± 3.6%, 2.1 ± 0.9% and 0.3 ± 0.2% mutant mtDNA and 32.1 ± 3.2%, 98.9 ± 0.6% and 101.3 ± 1.3% wild-type mtDNA, respectively. To ensure that changes in heteroplasmy were maintained for longer periods of time, we performed another experiment where a cybrid harbouring approximately 90% mutant mtDNA was treated with mitoARCUS RNA and heteroplasmy analysed up to 42 days. These results showed that the change in heteroplasmy observed at days 2–3 was maintained over time (Supplementary Fig. 3).

Because mutations in mitochondrial tRNA^Leu(UUR)^ are expected to impair mitochondrial protein synthesis^49^, we measured the levels of two proteins dependent on mitochondrial protein synthesis: MT-CO1, which is encoded by mtDNA, and NDUFB8, which is encoded by nuclear DNA but is very unstable when its mtDNA-encoded partners in Complex I assembly are missing^50^. Immunoblots indicated that levels of both markers were increased 3 days following mitoARCUS3.2 mRNA nucleofection (Fig. 3b). Another important indicator of mitochondrial function is respiration, typically measured as oxygen consumption rate (OCR) at either baseline (basal respiration) or following the addition of an uncoupler to record maximal respiratory capacity (maximal respiration). When these cells were evaluated for respiration (Fig. 3c) we observed that all three heteroplasmy-shifted conditions had basal (Fig. 3d) and maximal (Fig. 3e) OCR values 1.8-fold higher than control. The observed shifts in heteroplasmy (Supplementary Fig. 4a) and increases in mitochondrial protein steady-state levels (Supplementary Fig. 4b) and respiration rate (Supplementary Fig. 4c–e) were sustained at day 7.

Notably, containing 68.7% mutant mtDNA demonstrated OCRs equivalent to those of cells containing 0.3% mutant mtDNA (Fig. 3c–e and Supplementary Fig. 4c–e), indicating that the threshold for exhibiting respiratory impairment in these cells exists somewhere between 68 and 95% mutant mtDNA. We sought to corroborate this threshold effect by evaluating functional outcomes of a heteroplasmy shift in a lower-percentage mutant cybrid line. mRNA encoding mitoARCUS3.2 or MTS-GFP was nucleofected into cell line B (85% mutant) at a dose of 1 × 10^5^ mRNA copies per cell. At day 3 no significant alterations in mtDNA copy number were observed in mitoARCUS3.2-treated cells yet the level of wild-type mtDNA present in these cells was 100.1 ± 0.3% (Supplementary Fig. 5a). Despite this shift in heteroplasmy, no significant changes in respiration were observed (Supplementary Fig. 5b–d), indicating that shifting heteroplasmy below 85% did not confer a functional benefit. Thus, levels of m.3243G mutant mtDNA greater than 85% are probably necessary to observe a defect in respiration in this cell culture system. These data support our initial observations and suggest that, for very-high-percentage mutant cells, such as cell line C, small shifts in heteroplasmy of only 5–10% could significantly improve mitochondrial function.

### Transient mtDNA depletion does not impact respiratory function in cybrids

While mitoARCUS-induced shifts in heteroplasmy were encouraging, it was conceivable that the shift from 95 to 0.3% mutant mtDNA in 3 days could be accompanied by detrimental mtDNA depletion between days 0 and 3. This was concerning, because transient mtDNA depletion could result in undesirable impairment in mitochondrial respiratory function. To test this possibility, mitoARCUS3.2 mRNA or control mRNA (MTS-GFP) was nucleofected into cell line C at a dose of 1 × 10^5^ mRNA copies per cell. At day 1 post transfection, cellular DNA was collected for heteroplasmy and mtDNA copy number analysis and live cells were analysed for respiration.

**Fig. 4:**
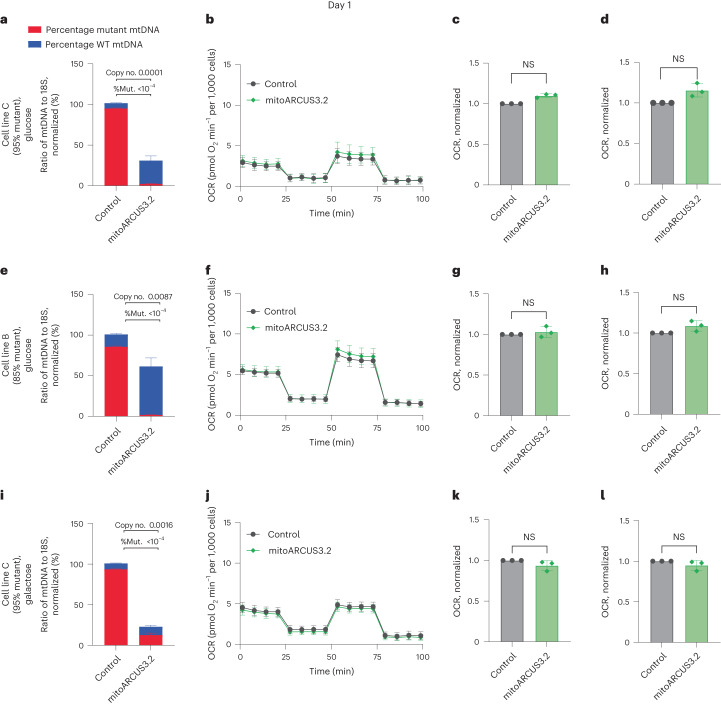
Transient mtDNA depletion induced by mitoARCUS3.2 does not impact respiration in high-percentage m.3243G mutant cybrid cells grown in glucose or galactose. **a**–**l**, Cell lines C (95% mutant) and B (85% mutant) were nucleofected with mRNA encoding either MTS-GFP or mitoARCUS3.2 at a dose of 1 × 10^5^ mRNA copies per cell. Cellular DNA was collected at day 1 for mtDNA heteroplasmy and mtDNA copy number analysis, and live cells were analysed for respiration. mtDNA heteroplasmy, normalized to mtDNA copy number of control in cell line C (95% mutant) cultured in glucose-containing medium (**a**). Seahorse Cell Mito Stress Test for cells described in **a** (**b**). Basal respiration for cells described in **a**, normalized to control (**c**). Maximal respiration for cells described in **a**, normalized to control (**d**). mtDNA heteroplasmy, normalized to mtDNA copy number of control in cell line B (85% mutant) cultured in glucose-containing medium (**e**). Seahorse Cell Mito Stress Test for cells described in **e** (**f**). Basal respiration for cells described in **e**, normalized to control (**g**). Maximal respiration for cells described in **e**, normalized to control (**h**). mtDNA heteroplasmy, normalized to mtDNA copy number of control in cell line C (95% mutant) cultured in galactose-containing medium (**i**). Seahorse Cell Mito Stress Test for cells described in **i** (**j**). Basal respiration for cells described in **i**, normalized to control (**k**). Maximal respiration for cells described in **i**, normalized to control (**l**). Data encompass three independent experiments and are shown as mean ± s.d. Statistical analysis was performed using a two-tailed *t*-test on raw data. NS, *P* > 0.05; all other *P* values indicated. Source data

As expected, the high mitoARCUS mRNA dose generated a transient depletion in mtDNA to approximately 30% of control at day 1 (Fig. 4a). This mtDNA depletion coincided with the specific elimination of mutant mtDNA genomes, because there was only 7.3 ± 4.6% mutant mtDNA present while wild-type mtDNA constituted 91.6 ± 4.3% of the remaining mtDNA. Despite partial mtDNA depletion, respiration was not negatively impacted (Fig. 4b–d).

Due to the overall low level of respiration observed in cell line C (Supplementary Fig. 2a–c), we reasoned that a respiratory defect due to partial mtDNA depletion might not be observable. Therefore, the experiment described above was repeated in cell line B (85% mutant), which possessed a level of mutant mtDNA below the mutation threshold necessary for detection of phenotypic impairments, and thus we reasoned that any respiratory impediment due to mtDNA depletion would be more likely to be noticeable. At day 1 partial mtDNA depletion was observed (Fig. 4e) although there were no significant changes in respiration (Fig. 4f–h), indicating that transient mtDNA depletion had had no measurable impact on mitochondrial function in these cells.

The experiments described above were performed with cybrid cell lines cultured in high-glucose-containing medium, which allowed them to rely mainly on glycolysis for ATP production^51^. However, cells in vivo, particularly high-energy-demand cells that are preferentially impacted in MELAS, would rely heavily on mitochondrial respiration for ATP generation and survival^52^. Therefore, we sought to make the cell culture environment more comparable to an in vivo context by forcing cells in culture to rely on OXPHOS for ATP production. By transitioning cells from glucose- to galactose-containing medium, cellular reliance is switched to OXPHOS for ATP production because there is no net gain in ATP from the oxidation of galactose to pyruvate^53,54^. Thus, pyruvate must be fully oxidized through mitochondrial respiration to generate ATP. Once again, mRNA encoding mitoARCUS3.2 or MTS-GFP was nucleofected into cell line C (95% mutant), this time with cells cultured in galactose-containing medium following transfection. At day 1 again, partial, transient mtDNA depletion was observed (Fig. 4i) that did not lead to any significant changes in respiration (Fig. 4j–l). At day 3 the mtDNA copy number of mitoARCUS-treated cells was recovered (Supplementary Fig. 6a) and a significant improvement in respiration was observed (Supplementary Fig. 6b–d), indicating that the transient mtDNA depletion observed at day 1 had not been detrimental to cells even when they were forced to rely on mitochondrial OXPHOS for ATP production.

### NES eliminates nuclear off-target editing

In addition to our focus on mtDNA specificity, we also explored the effects of mitoARCUS3.2 treatment on nuclear off-target editing. As shown in Fig. 1b, nucleofecting ARCUS mRNA lacking a targeting sequence resulted in some degree of nuclear localization. While this did not appear to occur when an MTS was incorporated into the construct (Fig. 1c), we sought to understand whether nuclear localization and off-target editing would be a concern in regard to mitoARCUS3.2—as has been shown to be for other mitochondrial gene-editing technologies^55,56^. We first sought to identify potential nuclease-induced nuclear off-target sites using an assay known as oligo capture, which utilizes a double-stranded DNA oligo tag containing randomized 4 bp 3ʹ overhangs specifically designed to insert at sites of ARCUS-induced DSBs^47^. Following oligo insertion, cleaved sequences can be identified by next-generation sequencing (NGS). In this case a SV40 nuclear localization signal (NLS), rather than the MTS, was fused at the N terminus of ARCUS. By intentionally trafficking the protein to the nucleus we sought to identify any sequences that were at risk of being cleaved by a mislocalized ARCUS3.2. This assay utilized a human HEK 293 cell line modified to contain the mitoARCUS binding site on nDNA as an on-target control. Cells were cotransfected with a high dose of NLS-ARCUS3.2 mRNA (1 × 10^6^ mRNA copies per cell) along with a saturating dose of the dsDNA oligo tag. Two days following transfection, cellular DNA was collected and used to identify sites that were cleaved. This assay identified 226 potential nuclear off-target sites cleaved by the candidate enzyme in at least two replicates with either six or fewer mismatches or more than 1% of the number of reads recovered compared with the intended target site (Supplementary Fig. 7a). Of the 226 potential off-target sites, 14 were chosen for further analysis (Supplementary Table 1). The 14 sites chosen included nine out of the top ten sites based on number of reads recovered. While an increased number of reads generally correlates with increased off-target editing, this assay was not intended to be quantitative and further characterization would be required to quantify off-target editing.

Following identification of the top 14 potential nuclear off-target sites cleaved by NLS-ARCUS3.2, we sought to demonstrate the reliability of the oligo capture method in detection of potential off-target sites. By localizing the protein again to the nucleus, this time without cotransfection with the dsDNA oligo, the frequency of insertion/deletion (indel) formation induced by the nuclease at the introduced nuclear on-target and each off-target site could be quantified. NLS-ARCUS3.2 mRNA was transfected into the same modified HEK 293 cell line described above, this time at one of four different doses: 1 × 10^4^, 1 × 10^5^, 1 × 10^6^ or 2 × 10^6^ mRNA copies per cell. Cells were collected for analysis at day 6 post transfection. Editing at the introduced on-target sequence increased in a dose-dependent manner, reaching a maximum of 6.3 ± 1.2% (Supplementary Fig. 7b). Nuclear off-target editing was observed at 13 of the 14 identified off-target sites, starting at a dose of 1 × 10^5^ mRNA copies per cell and increasing as mRNA dose increased (Supplementary Fig. 7c). These data indicated that the off-target sites identified by oligo capture were true nuclease off-target sites that had been cleaved in a dose-dependent manner following ARCUS3.2 localization to the nucleus.

**Fig. 5:**
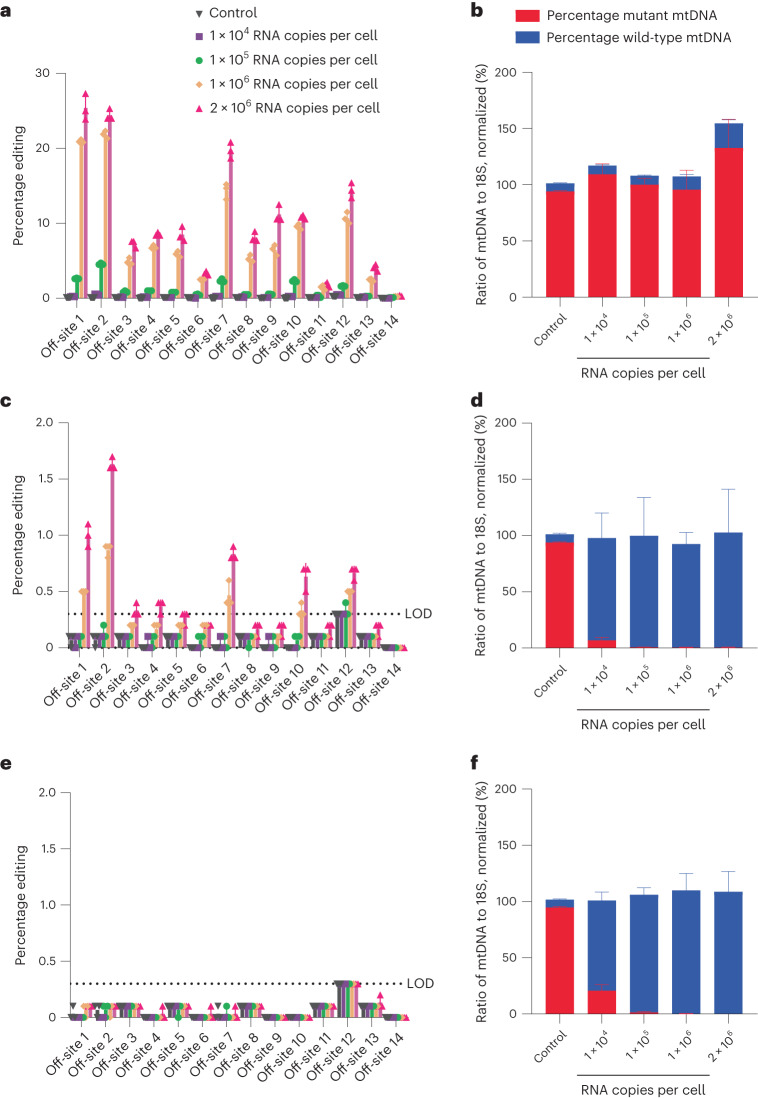
Incorporation of an MTS and NES into ARCUS3.2 eliminates nuclear off-target editing. **a**–**f**, ARCUS3.2 mRNA was nucleofected into cell line C (95% mutant) at various mRNA doses, ranging from 1 × 10^4^ to 2 × 10^6^ mRNA copies per cell. mRNA contained an NLS, MTS or both MTS and NES. Editing at each of the identified off-target sites, as well as an m.3243A>G heteroplasmy shift, was quantified at day 6 post transfection. Limit of detection (LOD) for off-target analysis was 0.3%. Indels generated at each of the 14 identified off-target sites by NLS-ARCUS3.2 (**a**). mtDNA heteroplasmy, normalized to mtDNA copy number of control, of cells treated with NLS-ARCUS3.2 (**b**). Indels generated at each of the 14 identified off-target sites by MTS-ARCUS3.2 (**c**). mtDNA heteroplasmy, normalized to mtDNA copy number of control, of cells treated with MTS-ARCUS3.2 (**d**). Indels generated at each of the 14 identified off-target sites by MTS-ARCUS3.2-NES (**e**). mtDNA heteroplasmy, normalized to mtDNA copy number of control, of cells treated with MTS-ARCUS3.2-NES (**f**). Data encompass three independent experiments and are shown as mean ± s.d. Source data

Following verification of editing at the identified off-target sites in the modified HEK 293 cell line, we sought to quantify nuclear off-target editing in cell line C (95% mutant). This cell line was chosen due to the presence of the on-target (mtDNA) sequence, thus allowing for evaluation of experimental efficacy via shifts in heteroplasmy when ARCUS3.2 was delivered with an MTS. First, NLS-ARCUS3.2 mRNA was transfected at the four doses described above to bridge between the two cell lines. Nuclear off-target editing was again observed at 13 of the 14 identified off-target sites (Fig. 5a), starting at a dose of 1 × 10^5^ mRNA copies per cell. Despite the nuclear localization of ARCUS, a slight shift in heteroplasmy was observed at the highest mRNA dose (Fig. 5b).

Next, mitoARCUS3.2 (MTS-ARCUS3.2) mRNA was transfected into cell line C at the doses described above. In this case, nuclear off-target editing was observed starting at 1 × 10^6^ mRNA copies per cell (Fig. 5c), a tenfold higher dose than that observed with NLS-ARCUS3.2. Importantly, heteroplasmy was shifted almost entirely (7.1 ± 0.1% mutant remaining) at the lowest evaluated dose of 1 × 10^4^ mRNA copies per cell (Fig. 5d), and it was not until the dose was increased 100-fold to 1 × 10^6^ mRNA copies per cell that nuclear off-target editing occurred. Nonetheless, the same sites that exhibited nuclear off-target editing with NLS-ARCUS3.2 also showed this with MTS-ARCUS3.2, although editing reached a maximum of only 1.5 ± 0.1% at the highest mRNA of 2 × 10^6^ mRNA copies per cell at the most abundantly cleaved off-target site.

Based on heteroplasmy shift data corresponding to each dose, we believed that nuclease specificity appeared to be dose responsive, suggesting a nuclease threshold for in vivo exposure to maintain specificity. To determine whether the inclusion of a NES could mitigate the observed nuclear off-target editing, the experiment described above was repeated, with a C-terminal NES fused to mitoARCUS3.2 (MTS-ARCUS3.2-NES). In this case nuclear off-target editing was not detected, even at the highest dose of 2 × 10^6^ mRNA copies per cell (Fig. 5e), while on-target efficacy remained unchanged (Fig. 5f). Therefore, it appears that nuclear off-target editing by mitoARCUS can be circumvented by inclusion of both an MTS and an NES on the construct.

### mitoARCUS shifts heteroplasmy in a m.3243A>G xenograft mouse model

At present there are no available animal models for the m.3243A>G mtDNA mutation. Therefore, we sought to generate a model to test a mitoARCUS therapeutic approach for shifting m.3243A>G heteroplasmy in vivo. Because the cybrid cells utilized here were derived from an osteosarcoma cell line, they are capable of tumour formation when injected in vivo. While a tumour is not a therapeutically relevant tissue, we reasoned that we could evaluate the tumour for evidence of heteroplasmy shift following systemic injection of AAV9-mitoARCUS into mice, thus generating in vivo proof of concept for this approach.

The xenograft model was generated by subcutaneous injection of cell line C (95% mutant) into the right flank of nude mice. Unlike many xenograft studies that seek to shrink the tumour following treatment, we did not expect mitoARCUS editing to impact tumour size. However, because there were restrictions regarding the volume to which tumours could reach, determining a cell dose that resulted in moderate, yet consistent, tumour growth was imperative to allow sufficient time for AAV treatment. A dose of 5 × 10^4^ cells in a total injection volume of 200 µl (Supplementary Fig. 8a) was chosen based on the consistency of tumour generation across pilot studies (Supplementary Fig. 8). At day 18 post cell injection, AAV9-mitoARCUS was administered systemically via retro-orbital injection at a dose of either 5 × 10^12^, 1 × 10^13^ or 5 × 10^13 ^vg kg^−1^. PBS was used as an injection control. Tumour volumes at the time of AAV/PBS injection ranged from 47 to 217 mm^3^, and among the different cohorts were not statistically significant (Supplementary Fig. 8g). All animals were euthanized at day 35 and tissues collected for molecular analysis. Multiple sections of the tumour were taken to assess efficacy across the tissue.

**Fig. 6:**
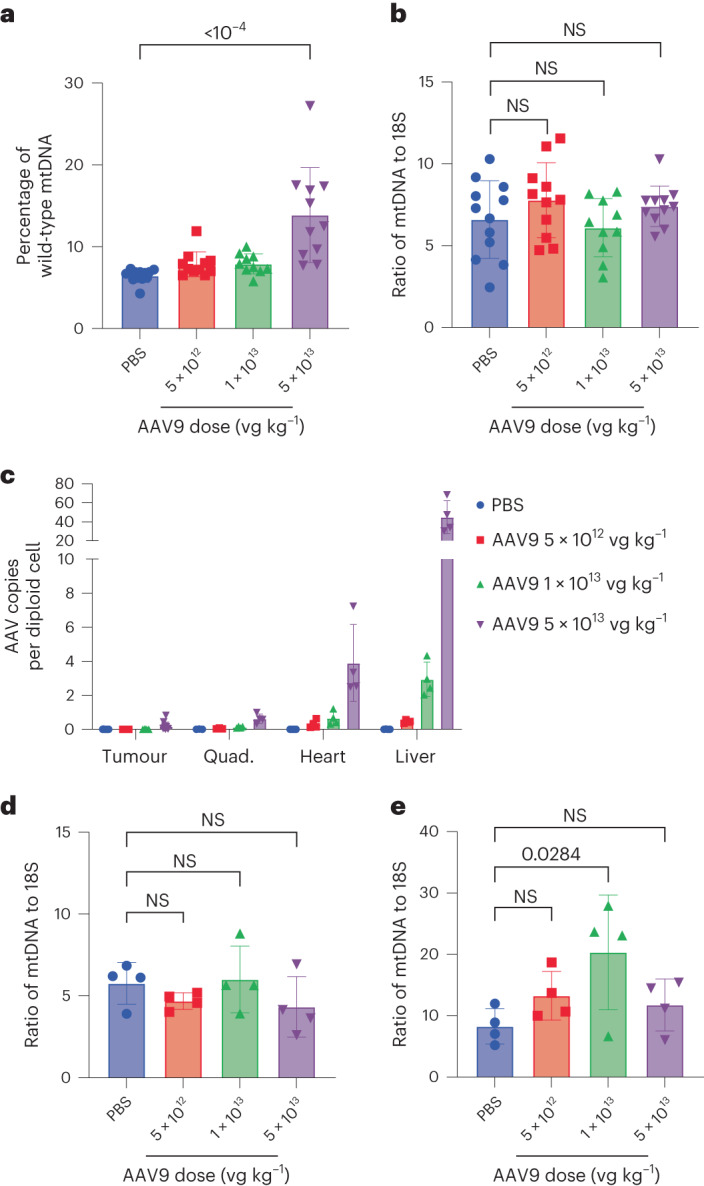
mitoARCUS shifts heteroplasmy in a xenograft mouse model following systemic AAV delivery, without mtDNA depletion. **a**–**e**, A xenograft mouse model with cells from cell line C (95% mutant) was used to evaluate mitoARCUS efficacy in vivo. AAV was used to deliver mitoARCUS systemically at various doses. Tumour, quadriceps (quad.), heart and liver were isolated at the conclusion of the study for molecular analysis. Percentage of wild-type mtDNA present in the tumour at study endpoint (day 35) (**a**). mtDNA copy number at day 35 in the tumour (**b**). AAV copy number across various tissues at day 35 (**c**). mtDNA copy number in the liver at day 35 (**d**). mtDNA copy number in the heart at day 35 (**e**). Four animals were included in each cohort, and three sections of each tumour were evaluated for heteroplasmy and mtDNA copy number. Data shown as mean ± s.d. Statistical analysis was performed using one-way ANOVA. NS, *P* > 0.05; all other *P* values indicated.

A dose-dependent shift in tumour mtDNA heteroplasmy was observed that reached statistical significance at the highest AAV9 dose (Fig. 6a). Tumours in the 5 × 10^13 ^vg kg^–1^ AAV9-treated cohort contained an average of 13.9 ± 5.8% wild-type mtDNA compared with 6.5 ± 0.8% in PBS-treated mice. There was no correlation observed between tumour volume at AAV injection and the resulting shift in heteroplasmy (*R* = −0.1434; Supplementary Fig. 8h). Additionally, no significant changes were observed in tumour mtDNA copy number for any of the AAV9-treated cohorts (Fig. 6b). Based on the impressive efficacy observed in cell culture, we hypothesized that the moderate heteroplasmy shift observed in vivo was probably due to low vascularity and/or transduction efficiency in the tumour, rather than to efficacy of the nuclease. To examine transduction efficiency we measured AAV copies per diploid cell in the tumour, quadriceps, heart and liver. Across all tissues we observed a dose-dependent increase in AAV copies per diploid cell (Fig. 6c). At an AAV9 dose of 5 × 10^13 ^vg kg^−^^1^ we observed 0.2 ± 0.2 copies per diploid cell in the tumour, 0.6 ± 0.3 in the quadriceps, 3.9 ± 2.3 in the heart and 45.0 ± 17.3 in the liver.

Due to the abundance of AAV copies found in both liver and heart, we evaluated mouse mtDNA copy number in these tissues to determine whether the human-specific mitoARCUS enzyme would demonstrate mouse mtDNA off-target cleavage. Despite the plethora of AAV copies there was no significant mtDNA depletion in either liver (Fig. 6d) or heart (Fig. 6e) in any of the AAV9-treated cohorts. These data support the specificity of the nuclease and indicate that, even at high doses, AAV9-mitoARCUS was not toxic to mice.

## Discussion

Although nuclease-mediated shifts in heteroplasmy have been demonstrated using mitochondrial-targeted restriction endonucleases^57–63^, mitochondrial-targeted TALENs (mitoTALENs)^36–41,64^ and mitochondrial-targeted ZFNs (mitoZFNs)^35,65–67^, each has disadvantages for therapeutic applications. The applicability of mitochondrial-targeted restriction endonucleases is limited based on mtDNA mutations that generate a novel restriction enzyme cut site^58^. MitoTALENs and mitoZFNs are both programmable, allowing for novel sequence recognition, but both are large, dimeric proteins that typically require two AAV vectors for in vivo delivery^39,67^. By contrast, mitoARCUS nucleases are only ~1,400 bp, including the MTS and NES, and therefore fit comfortably within the 4.5 kb packaging limit of single-stranded AAV. The need for only one AAV vector and the single-component architecture greatly simplify manufacturing and delivery for clinical applications, thus making mitoARCUS an attractive alternative to the more commonly used mitoTALENs and mitoZFNs. However, the protein engineering required to re-engineer I-*Cre*I to recognize novel target sequences presents a technical challenge. While theoretically a new enzyme could be designed to target any 22 bp DNA sequence, the middle 4 bp must bend to allow proper positioning of the active site. This is best achieved in recognition sequences where a pyrimidine is followed by a purine at the two central bases^68^. This requirement somewhat limits the sequences that can exist in those positions. Additionally, each new target site requires a novel enzyme^42,44–48^.

The primary concern with any in vivo gene-editing therapeutic is specificity. In the context of heteroplasmic mtDNA mutations, nuclease specificity for the mutant mtDNA sequence is imperative because there is only a single nucleotide difference between the mutant and wild-type sequences. However, it is possible that other off-target sequences could exist within the mtDNA. If this did occur, the outcome of that off-target mtDNA cleavage would be the same as cleavage at the wild-type version of the recognition sequence—mtDNA depletion. Therefore, while presently we do not have a method to identify specific sites of mitoARCUS-induced DSB in the mtDNA, we can still detect these events by measuring mtDNA copy number in homoplasmic wild-type cells, as demonstrated in Fig. 2c. For the doses evaluated here we did not detect significant depletion of wild-type mtDNA at any of the analysed time points with mitoARCUS3.2, indicating a lack of wild-type mtDNA cleavage and therefore a high degree of specificity of this enzyme. This strict specificity allowed for robust shifts in heteroplasmy across a 2,000-fold difference in mRNA dose without jeopardizing wild-type mtDNA molecules (Figs. 3a and 5d,f). Importantly, the doses used for evaluation of nuclease specificity (Fig. 2c,d and Supplementary Fig. 1a,b) occurred within this range. The broad dynamic range of this highly specific nuclease will probably allow for flexibility in terms of in vivo dosing and efficacy without impacting wild-type mtDNA molecules.

These mitoARCUS-induced heteroplasmy shifts were found to generate significant improvements in both basal and maximal respiration at 3 days post transfection in a cell line harbouring 95% m.3243G mutant mtDNA. These shifts in heteroplasmy were maintained at day 7 and beyond 1 month (Supplementary Figs. 3 and 4). Notably, we observed a strong threshold effect where cells containing 68.7% mutant mtDNA respired equivalently to those containing 0.3% mutant mtDNA, indicating that a complete shift to 100% wild-type mtDNA is not necessary to achieve meaningful phenotypic improvements. This finding is particularly impactful when considering the therapeutic potential of nuclease-mediated shifts in heteroplasmy: the percentage of mutant mtDNA probably just needs to fall below the biochemical threshold to achieve the desired outcome.

Despite these robust shifts in heteroplasmy and improvements in respiration, we were concerned that the mtDNA depletion that must precede a complete shift in heteroplasmy could be problematic in regard to mitochondrial function. The data described here suggest that this is not the case, and is probably due to a lack of functionality of the mutant mtDNA molecules being eliminated as well as the extended half-lives of OXPHOS complex proteins^69^. However, this should be evaluated in more relevant model systems. Additionally, our proposed in vivo delivery method would be by AAV, which will probably generate a longer-term and more stable level of nuclease expression compared with the transient bolus generated by mRNA delivery. Therefore, as previously observed for other mitonucleases, including mitoARCUS^42^, AAV delivery of the nuclease would probably generate more gradual shifts in heteroplasmy than we have observed in cell culture, thus avoiding the rapid mtDNA depletion observed with transient mRNA delivery.

Previous studies with mitoARCUS delivered by AAV in vivo did not detect any nuclear off-target editing^42^. However, the cell culture experiments described here found up to 1.5 ± 0.1% nuclear off-target editing at the most frequently cut off-target site, without an NES included on the construct. This led us to believe that the nuclear off-target editing we observed may be an artefact of the cell culture model and transfection system. We used nucleofection to introduce mRNA into cells, which permeabilizes not only the plasma membrane but also the nuclear pores, thus making them more amenable to uptake of molecules^70^. Additionally we utilized high doses of mRNA to deliver the payload, which generates a transient bolus of mitoARCUS protein that would be unlikely to occur following AAV delivery in vivo. However, the inclusion of an NES in mitoARCUS effectively eliminated the observed nuclear off-target editing in our cell culture system and therefore additional nuclease optimization was not completed.

The novel xenograft mouse model described here allowed for the evaluation of the in vivo efficacy of mitoARCUS using AAV as the delivery vehicle, without any overt signs of vehicle-related toxicity. However, transduction of the tumour was low when compared with more disease-relevant tissues, indicating that heteroplasmy shifts would probably be greater in these tissues if the mitoARCUS binding site sequence was present. The low level of tumour transduction could have been due to either low vascularization in the tumour, the tissue specificity of AAV9 or other reasons. However, the same AAV serotype was used in a m.5024C>T heteroplasmic mouse model to deliver a mitoARCUS enzyme specific for that mutation, and demonstrated significant shifts in heteroplasmy across a number of tissues including liver, quadriceps, tibialis anterior, gastrocnemius and kidney^42^. Together, these data suggest that the MELAS-specific mitoARCUS enzyme could be expected to be delivered to the same disease-relevant tissues in vivo using AAV9.

Of relevance to many mitochondrial diseases, including MELAS, is the ability to target cells of the central nervous system (CNS), particularly motor neurons^71^. Successful delivery to the CNS has been demonstrated following intravenous injection of AAV9 in mice^72^, non-human primates^73,74^ and humans^75,76^. However, targeting of motor neurons was most effective following early postnatal administration^77^. Unless prenatal or newborn screening for this mtDNA mutation is utilized, it may not be feasible to achieve sufficient CNS tissue transduction following systemic administration. Alternatively, injection of AAV9 directly into the cerebral spinal fluid of mice and non-human primates demonstrated the same level of motor neuron targeting as a tenfold higher dose delivered systemically^78^. Therefore, an AAV9-mitoARCUS gene therapy could be developed for patients with the m.3243A>G mutation and the route of administration could differ based on age and disease presentation. In summary, we believe that mitochondrial diseases caused by heteroplasmic mtDNA point mutations are an area of high unmet need and that mitoARCUS fills a unique niche among the various gene-editing technologies for the treatment of these diseases. Additionally, the data presented here offer promise for patients afflicted with m.3243A>G-associated diseases.

## Methods

### Nuclease development and optimization

A library of nucleases designed to cleave m.3243G mutant mtDNA was designed and selected using a proprietary directed evolution system as previously described^47^. The 22 bp target sequence surrounding m.3243G that was targeted was 5'-CAGGGCCCGGTAATCGCATAAA-3'. The nuclease selection process involved both a positive selection (ability of nucleases to cut the on-target (mutant) sequence) and a negative selection (ability of the nucleases not to cut the off-target (wild-type) sequence). The resulting nucleases were screened in a high-throughput GFP assay in Chinese hamster ovary cells as previously described^42^. Nucleases that generated high GFP expression in the mutant cell line and low GFP expression in the wild-type cell line were selected for further analysis. The first round of nuclease optimization involved the modification of various amino acids in mitoARCUS1.1. Ten amino acids (positions 46, 75, 77, 182, 219, 229, 233, 259, 263 and 271) were changed to generate mitoARCUS2.1. The second round of nuclease optimization involved further modification of the amino acids in mitoARCUS2.1 to generate mitoARCUS 3.1 and mitoARCUS 3.2. Both mitoARCUS3.1 and mitoARCUS3.2 differed at six amino acids from mitoARCUS 2.1 (positions 19, 70, 75, 80, 259 and 263 and positions 19, 46, 70, 75, 80 and 263 for mitoARCUS3.1 and mitoARCUS3.2, respectively). mitoARCUS3.1 and mitoARCUS3.2 differed from each other at only two amino acids (positions 46 and 259). Position 19 is involved in positioning of the metal ion and impacts enzyme activity, and positions 80 and 271 are backbone contacts that influence general DNA affinity; the remaining positions are involved in direct nucleotide contacts.

### Generation of cybrid cell lines

A cybrid cell line (based on 143B human osteosarcoma, ATCC CRL-8303) containing 78% m.3243G mutant mtDNA was treated with 50 ng ml^−^^1^ ethidium bromide for 15 days to substantially deplete mtDNA copy number, and the resulting clones were isolated using ring cloning^79^. This produced clones with 100, 95 and 85% m.3243G mutant mtDNA. The 0% m.3243G mutant cell line was generated by treating the parent cell line with mitoARCUS.

### Cybrid cell culture and transfection

Cybrid cells were maintained in DMEM (Thermo Fisher) with 10% fetal bovine serum (FBS; Thermo Fisher), 1 mM sodium pyruvate (Thermo Fisher), 50 µg ml^−^^1^ uridine (MilliporeSigma), 20 µg ml^−^^1^ gentamycin (Thermo Fisher) and 5 µg ml^−^^1^ Plasmocin (InvivoGen) and grown in 5% CO_2_, 37 °C humidity-controlled incubators. For experiments utilizing galactose rather than glucose, cells were grown in DMEM with no glucose (Thermo Fisher) supplemented with 5 mM galactose (Sigma-Aldrich), 10% FBS, 1 mM sodium pyruvate, 50 µg ml^−^^1^ uridine, 20 µg ml^−^^1^ gentamycin and 5 µg ml^−^^1^ plasmocin. All cybrids were transfected using a Lonza 4D-Nucleofector (Lonza Bioscience, SF buffer, condition CA-137). In total, 8 × 10^5^ cells were nucleofected per condition.

### mitoARCUS construct design

Each mitoARCUS enzyme contained an N-terminal MTS comprising COX8A and SU9: N- MSVLTPLLLRGLTGSARRLPVPRAKIHSLPPEGKLMASTRVLASRLASQMAASAKVARPAVRVAQVSKRTIQTGSPLQTLKRTQMTSIVNATTRQAFQKRAYSS-C^39,42^. When an NES sequence was used, this was added at the C terminus of the enzyme and was derived from the NS2 proteins of parvovirus minute virus of mice (MVMp NS2): N-VDEMTKKFGTLTIHDTEK-C^66,80^. For nuclear off-target editing, an N-terminal NLS from SV40 replaced the MTS: N-MAPKKKRKVH-C.

### mRNA generation

The nucleases described above were cloned into a plasmid vector containing a T7 promoter, 5' and 3' untranslated regions and a polyT repeat to serve as a template for a >100 bp polyA tail. The DNA template was linearized immediately following the polyT tail, and the product purified using NucleoSpin Gel and PCR Clean-up Columns (Macherey-Nagel). In vitro mRNA transcription was performed using a HiScribe T7 High Yield RNA Synthesis Kit (New England Biolabs), substituting with 2.5 mM N1-methyl-pseudouridine-modified NTP (TriLink BioTechnologies) and 5 mM CleanCap AG (TriLink). DNase treatment was performed according to the manufacturer’s protocol and RNA was purified using the SV Total RNA Isolation System (Promega). RNA concentrations were determined by ultraviolet absorption using a NanoDrop Spectrophotometer (Thermo Fisher), and RNA quality was assessed on a 5200 Fragment Analyzer (Agilent Technologies).

### Fluorescence microscopy

Cell line C was nucleofected with mRNA encoding a mitoARCUS-GFP fusion. At day 1, cells were stained with 50 nM MitoTracker Deep Red FM (Thermo Fisher) and Hoechst 33342 (Thermo Fisher) at 1:5,000 dilution in standard cell culture medium. Live cells were used for fluorescence microscopy with a ZEISS Axio Observer and images processed with deconvolution using Zen 3.4 (ZEISS).

### Cellular DNA extraction

Cellular DNA was isolated from cultured cells and mouse tissue using the NucleoSpin Blood QuickPure kit (Macherey-Nagel). DNA concentrations were obtained using a Lunatic UV/Vis absorbance spectrometer (Unchained Labs).

### Quantification of mtDNA copy number and mtDNA linearization

mtDNA copy number was quantified as previously described using duplex direct PCR (dPCR), with one assay that amplified *MT-ND2* and another that amplified 18S rDNA^81^. Primers are given in Supplementary Table 2. The ratio of the two assays provided a ratio of mtDNA copy number relative to 18S rDNA. mtDNA linearization was quantified using one dPCR assay that amplified *MT-TL1*, specifically spanning the m.3243 heteroplasmic base, and the same *MT-ND2* assay described above. The ratio of the two assays provided a ratio of circular mtDNA genomes at position m.3243 relative to total mtDNA genomes.

### Simultaneous quantification of m.3243A>G heteroplasmy and mtDNA copy number

mtDNA heteroplasmy and copy number were quantified by duplex dPCR as previously described^81^. The calculated mtDNA copy number for each treated condition was normalized to control at each timepoint. The resulting normalized copy number value was then multiplied by the heteroplasmy level to generate the combined heteroplasmy/copy number data found in Figs. 3, 4 and 5 and Supplementary Figs. 4, 5 and 6.

### Mitochondrial protein steady-state levels

Protein lysates were prepared from flash-frozen cell pellets following sonication. Total protein concentration was quantified using a Pierce BCA Protein Assay Kit (Thermo Fisher). Total protein per sample (30 µg) was run in NuPAGE 10% Bis-Tris 1.0–1.5 mm mini protein gels (Invitrogen) and then transferred onto polyvinylidene difluoride membranes (Invitrogen) using an XCell SureLock Mini-Cell (Invitrogen). Blots were blocked for 1 h at room temperature with 5% non-fat dry milk dissolved in Tris buffered saline with Tween. Primary antibodies used were mouse monoclonal anti-MT-CO1 (1D6E1A8, no. ab14705, Abcam, 1:1,000 dilution), mouse monoclonal anti-NDUFB8 (20E9DH10C12, no. ab110242, Abcam, 1:1,000 dilution) and mouse monoclonal anti-alpha-tubulin (DM1A, Loading Control, no. ab7291, Abcam, 1:20,000 dilution). The secondary antibody was goat anti-mouse IgG H&L (horseradish peroxidase, Abcam, 1:5,000 dilution). Primary antibodies were incubated overnight at 4 °C and the secondary antibody was incubated for 1 h at room temperature. Membranes were developed with Amersham ECL Prime Western Blotting Detection Reagent (Cytiva) and imaged using G:Box F3 (Syngene).

### OCR rates

Oxygen consumption rates were measured using the Seahorse XFe96 Analyzer (Agilent Technologies). For cells in glucose, 4 × 10^3^ cells per well were plated ~18 h before beginning the assay in standard cell culture medium in a total volume of 80 µl. Cells were stored at room temperature for 1 h before overnight storage in an incubator at 5% CO_2_ and 37 °C. A XFe96 sensor cartridge was hydrated with Seahorse XF Calibrant overnight in a non-CO_2_ 37 °C incubator. Before assay, cells were washed two times with Seahorse Assay Medium (DMEM) containing 1 mM sodium pyruvate, 2 mM glutamine and 10 mM glucose (Agilent Technologies) and then equilibrated in a non-CO_2_ 37 °C incubator for 1 h. The One Cell Mito Stress Test kit (Agilent Technologies) was reconstituted according to the manufacturer’s recommendations. Working stocks were generated for each of the compounds as follows: 15 µM oligomycin, 5 µM carbonyl cyanide 4-(trifluoromethoxy) phenylhydrazone (FCCP) and 5 µM rotenone/antimycin A. These solutions were added to ports A, B and C of the XFe96 sensor cartridge as follows: 20 µl of oligomycin (port A), 22 µl of FCCP (port B) and 24 µl of rotenone/antimycin A (port C). Four readings were collected for each phase of the assay. OCR values were normalized to cell count per well using image cytometry (Molecular Devices) at completion of the assay, and data analysis was conducted using Wave software (Agilent Technologies).

For cells in galactose the protocol was the same apart from the following exceptions. Cells were plated at a seeding density of 7.5 × 10^3^ per well in DMEM no glucose (Thermo Fisher) with 5 mM galactose (Sigma-Aldrich). The Seahorse Assay Medium (DMEM) was supplemented with 10 mM galactose (Sigma-Aldrich) in place of glucose. The FCCP working stock was made up at 20 µM.

Basal respiration values were calculated by averaging OCR values for each of the technical replicates across time points 1–4. Maximal respiration values were calculated by averaging OCR values for each of the technical replicates across time points 9–12.

### Nuclear off-target identification (oligo capture)

Flp-In HEK 293 (Thermo Fisher) cells were generated containing the mitoARCUS binding site integrated onto the nuclear genome, and were maintained in Eagle’s minimum essential medium (ATCC) supplemented with 10% FBS, 1% penicillin-streptomycin-glutamine (Thermo Fisher), 1% sodium pyruvate (Thermo Fisher), 1% HEPES (Thermo Fisher), 1% HT supplement (Thermo Fisher) and 125 µg ml^−^^1^ hygromycin B (Thermo Fisher). Cells were cotransfected with 0.75 µg of oligo pool and 1.5 µg of nuclease mRNA (1 × 10^6^ mRNA copies per cell) per 1.5 × 10^6^ cells using the Neon Transfection System (Thermo Fisher, 1,100 V pulse voltage, 40 ms pulse width, one pulse). Nuclease mRNA contained an NLS at the N terminus and each sample was transfected in triplicate. Cells were harvested at day 2 post electroporation for genomic DNA isolation, which was processed as previously described^47^. Potential nuclear off-target sites were identified by NGS at the site of oligo incorporation. Individual genomic sites with incorporated oligo are indicated as blue dots in Supplementary Fig. 7a, with the darker-coloured dots representing sites with greater sequence similarity compared with the intended target site, and lighter-coloured dots having more sequence variation. The read count indicated the number of unique reads aligned at each site location, with higher counts indicating a higher likelihood of nuclease-mediated off-target editing. The integrated on-target site is represented by a red dot.

### Nuclear off-target quantification

Modified Flp-In 293 cells (1.5 × 10^6^) were transfected with 1 × 10^4^, 1 × 10^5^, 1 × 10^6^ or 2 × 10^6^ mRNA copies per cell using the Neon Transfection System (Thermo Fisher, 1,100 V pulse voltage, 40 ms pulse width, one pulse). Nuclease mRNA contained an NLS at the N terminus. Cells were harvested at day 6 post electroporation for genomic DNA isolation, NGS analysis of nuclear off-target editing at identified sites and dPCR analysis of on-target editing at the introduced target site. On-target editing was quantified using duplex dPCR, with one assay that spanned the introduced binding site and another as a genomic reference. Primers are described in Supplementary Table 2. dPCR amplifications were duplexed in a 24 µl reaction containing 1× dPCR Supermix for Probes (no dUTP, Bio-Rad), 250 nM each probe, 900 nM each primer, 20 U µl^−^^1^ Mfe-I-HF and 150 ng of cellular DNA. Droplets were generated using a QX200 droplet generator (Bio-Rad) and PCR was performed on a C1000 Touch thermal cycler (Bio-Rad). Cycling conditions were as follows: one cycle at 95 °C (2 °C s^−^^1^ ramp) for 10 min, 45 cycles of 94 °C (2 °C s^−^^1^ ramp) for 10 s, 61.1 °C (2 °C s^−^^1^ ramp) for 30 s, 72 °C (0.2 °C s^−^^1^ ramp) for 150 s, one cycle of 98 °C for 10 min and 4 °C hold. Droplets were analysed using a QX200 droplet reader (Bio-Rad), and QuantaSoft analysis software (Bio-Rad) was used to acquire and analyse data. Editing was quantified by calculating the ratio of binding site (FAM)-positive droplets to reference (HEX)-positive droplets and subtracting that ratio for each treated sample from the control.

For nuclear off-target evaluation in cell line C (95% mutant), 8 × 10^5^ cells were transfected with 1 × 10^4^, 1 × 10^5^, 1 × 10^6^ or 2 × 10^6^ mRNA copies per cell using a Lonza 4D-Nucleofector (Lonza Bioscience, SF buffer, condition CA-137). Cells were harvested at day 6 post electroporation for genomic DNA isolation, NGS analysis of nuclear off-target editing at identified sites and dPCR analysis of mtDNA heteroplasmy and copy number as described above.

NGS analysis of indel frequencies at the 14 sites chosen from the oligo capture assay was performed using rhAmpSeq custom panels (Integrated DNA Technologies). Library preparation was performed according to manufacturer protocols using 200 ng of genomic DNA for each reaction. Libraries were sequenced using an Illumina sequencer with 2 × 150 bp reads, and indel frequencies for each amplicon were calculated using custom software as previously described^47^.

### AAV9 preparation

Expi293F cells were grown in Expi293 Expression Medium (Thermo Fisher) supplemented with penicillin-streptomycin-glutamine (Thermo Fisher) and were transfected using the triple-plasmid cotransfection system with polyethylenimine hydrochloride (Polysciences). Cells were lysed 72 h post transfection using 50 mM Tris, 1% polysorbate 20, 2 mM magnesium chloride and 400 mM sodium chloride. Lysed cells were incubated at 37 °C for 30 min with agitation. After lysis, 100 U ml^−^^1^ Salt Active Nuclease (ArcticZymes) was added and lysates were incubated for 90 min at 37 °C with agitation, followed by the addition of 50 mM EDTA and incubation at 37 °C for 15 min. The lysate was then centrifuged for 1 h at 3,000*g* and the supernatant passed through a Supor EKV 0.2 µM filter (Pall Corporation). To further purify AAV, the filtrate was subjected to affinity chromatography using POROS CaptureSelect AAVX Affinity Resin (Thermo Fisher) followed by anion-exchange chromatography using a CIMmultus QA monolithic column (Sartorius). The eluate containing purified AAV was buffer exchanged into PBS + 0.001% Pluronic F-68 (Thermo Fisher).

### Xenograft mouse model

The xenograft mouse model was generated using 7–9-week-old homozygous J:Nu mice obtained from The Jackson Laboratory (stock no. 007850). Mice (Nu/Nu females, 9–11 weeks old) were injected subcutaneously in the right flank with 5 × 10^4^ cells from cell line C mixed in a 1:1 ratio of PBS and growth-factor-reduced, phenol red-free Matrigel (Corning Life Sciences). Once palpable, emerging tumours were measured biweekly via digital calipers. At day 18, mice were injected in the retro-orbital space with either PBS or AAV9-mitoARCUS. The mitoARCUS construct used contained an MTS, but not an NES. All mice were humanely euthanized at day 35, once tumours had reached 2,000 mm^3^ (the size limit allowed by the Institutional Animal Care and Use Committee protocol). Animal experiments were approved by the Institutional Animal Care and Use Committee of Mispro Biotech and adhered to the National Institutes of Health guide for the care and use of laboratory animals. Light cycles were held constant at a 12/12-h light/dark cycle and food and water were administered ad libitum. Temperatures in mouse rooms were kept at 20–25 °C with 40–60% humidity.

### Quantification of AAV copy number

AAV copy number across various tissues was quantified using duplex dPCR, with one assay that amplified AAV DNA and another that amplified nDNA (either mouse or human; primers described in Supplementary Table 2). dPCR amplifications were duplexed in a 24 µl reaction containing 1× dPCR Supermix for Probes (no dUTP, Bio-Rad), 250 nM each probe, 900 nM each primer, 20 U µl^−^^1^ Hind-III-HF and cellular DNA (1.8 ng for mouse tissue, 90 ng for human tumour). Droplets were generated using a QX200 droplet generator (Bio-Rad) and PCR was performed on a C1000 Touch thermal cycler (Bio-Rad). Cycling conditions were as follows: one cycle of 95 °C (2 °C s^−^^1^ ramp) for 10 min, 45 cycles of 94 °C (2 °C s^−^^1^ ramp) for 10 s, 57 °C (2 °C s^−^^1^ ramp) for 30 s, 72 °C (0.2 °C s^−^^1^ ramp) for 90 s, one cycle of 98 °C for 10 min and 4 °C hold. Droplets were analysed using a QX200 droplet reader (Bio-Rad), and QuantaSoft analysis software (Bio-Rad) was used to acquire and analyse data. AAV copy number per diploid cell was calculated by dividing the concentration (copies µl^−^^1^) of AAV DNA (HEX-positive) droplets by the concentration of nDNA (either mouse or human, FAM-positive) droplets and multiplying the resulting value by 2.

### Quantification of mouse mtDNA copy number

Mouse mtDNA copy number was quantified using duplex dPCR, with one assay that amplified MT-ND5 and another that amplified 18S rDNA. Primers are described in Supplementary Table 2. dPCR amplifications were duplexed in a 24 µl reaction containing 1× dPCR Supermix for Probes (no dUTP, Bio-Rad), 250 nM each probe, 900 nM each primer, 20 U µl^−^^1^ Hind-III-HF and ~18 pg of cellular DNA. Droplets were generated using a QX200 droplet generator (Bio-Rad) and PCR was performed on a C1000 Touch thermal cycler (Bio-Rad). Cycling conditions were as follows: one cycle of 95 °C (2 °C s^−^^1^ ramp) for 10 min, 45 cycles of 94 °C (2 °C s^−^^1^ ramp) for 10 s, 57 °C (2 °C s^−^^1^ ramp) for 30 s, 72 °C (0.2 °C s^−^^1^ ramp) for 90 s, one cycle of 98 °C for 10 min and 4 °C hold. Droplets were analysed using a QX200 droplet reader (Bio-Rad), and QuantaSoft analysis software (Bio-Rad) was used to acquire and analyse data. Mouse mtDNA copy number was calculated by dividing the concentration (copies µl^−^^1^) of mtDNA (HEX-positive) droplets by the concentration of mouse nDNA (FAM-positive) droplets.

### Statistical analysis

All data analysis was performed using GraphPad Prism 9. All statistics are presented as mean ± s.d. Pairwise comparisons were performed using an unpaired two-tailed *t*-test (NS, *P* > 0.05; all other *P* values indicated in the figures). Comparisons between more than two groups were done using one-way analysis of variance (ANOVA) and Dunnett’s multiple-comparisons test, comparing each treated group with control (NS, *P* > 0.05; all other *P* values are indicated in the figures). For any data shown as normalized, statistics were analysed using the raw data. Correlation was measured using Pearson’s correlation coefficient (*R*). No statistical methods were used to predetermine sample sizes, but our sample sizes are similar to those reported in previous publications^39,67^. Data distribution was assumed to be normal, but this was not formally tested. Mouse/samples were assigned to the various experimental groups randomly. Data collection and analysis were not performed blind to the conditions of the experiments. No animals or data points were excluded from analyses.

### Reporting summary

Further information on research design is available in the Nature Portfolio Reporting Summary linked to this article.

### Supplementary information

Supplementary Information Supplemental Figs. 1–8 and Tables 1 and 2.

Reporting Summary

### Source data

Source Data Fig. 3 Spreadsheet with the data used in graphs.

Source Data Fig. 3 Unprocessed immunoblots.

Source Data Fig. 4 Spreadsheet with the data used in graphs.

Source Data Fig. 5 Spreadsheet with the data used in graphs.

## Data Availability

Any data not contained in the paper are available on request with appropriate agreements. Source data are provided with this paper.
