# Supplementary material for: Efficient elimination of MELAS-associated m.3243G mutant mitochondrial DNA by an engineered mitoARCUS nuclease
Source: Nat Metab. 2023 Nov 30;5(12):2169–83. doi: 10.1038/s42255-023-00932-6 (PMC10730414; doi:10.1038/s42255-023-00932-6)
Supplement: Supplementary file 1 — Supplemental Figs. 1–8 and Tables 1 and 2. [file 42255_2023_932_MOESM1_ESM.pdf]

nature metabolism

Article

https://doi.org/10.1038/s42255-023-00932-6

Efficient elimination of MELAS-associated m.3243G mutant mitochondrial DNA by an engineered mitoARCUS nuclease

In the format provided by the authors and unedited

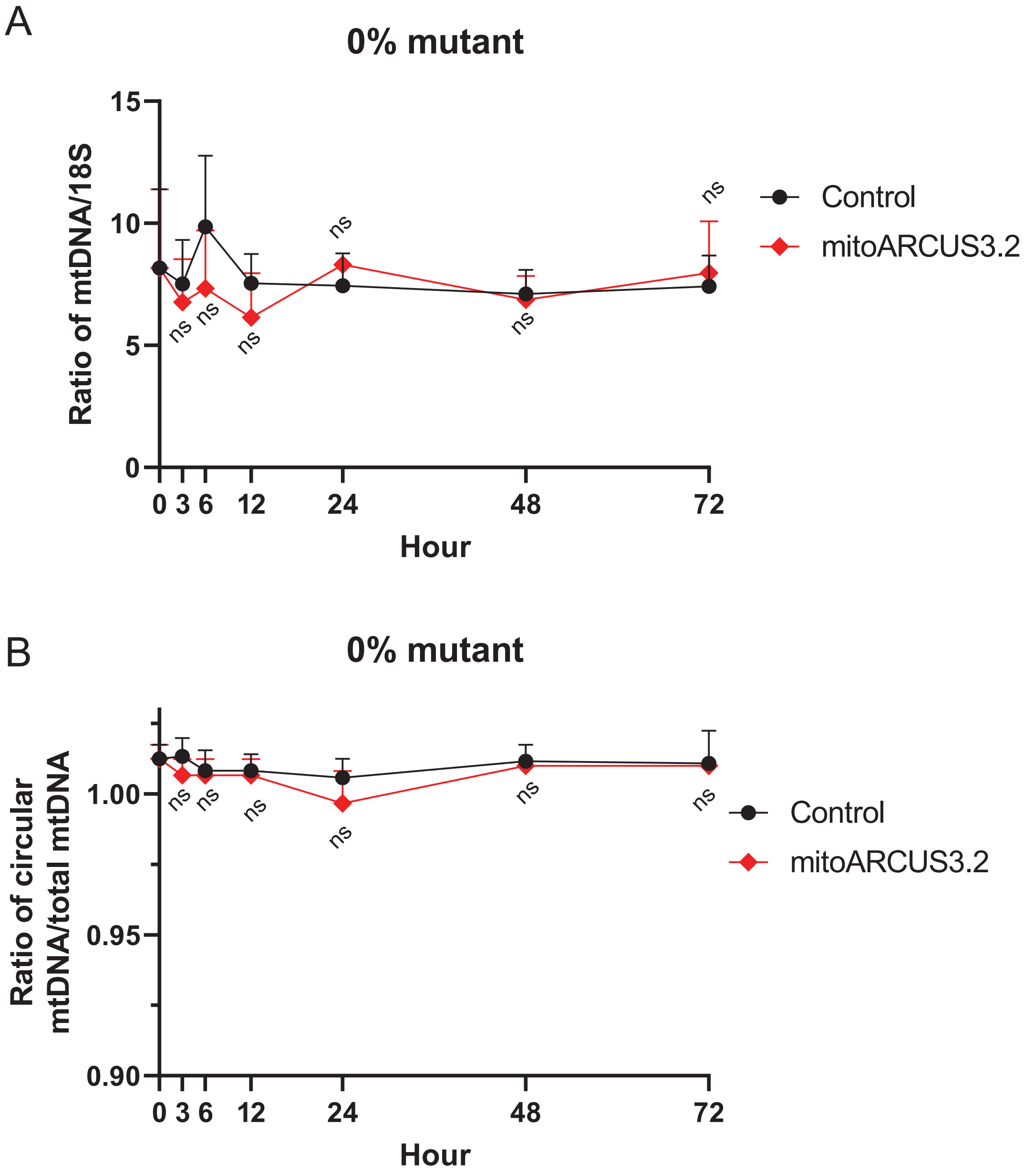

Supplemental Figure 1. mitoARCUS3.2 maintains specificity against wild-type m.3243A as mRNA dose is increased.
mitoARCUS3.2 was evaluated alongside an MTS-GFP control in cells containing 0% mutant (100% wild-type) mtDNA for mtDNA cleavage and loss. All mRNAs were nucleofected at a dose of 3x10□ mRNA copies/cell.

(A) mtDNA copy number over time in cell line A (0% mutant).
(B) mtDNA linearization at position m.3243 in cell line A (0% mutant).

Data encompass three independent experiments and are shown as mean + SD. Statistical analysis was performed using a two-tailed t-test. Ns: P>0.05.

mitoARCUS, mitochondrial-trafficked ARCUS; 18S, 18S rDNA.

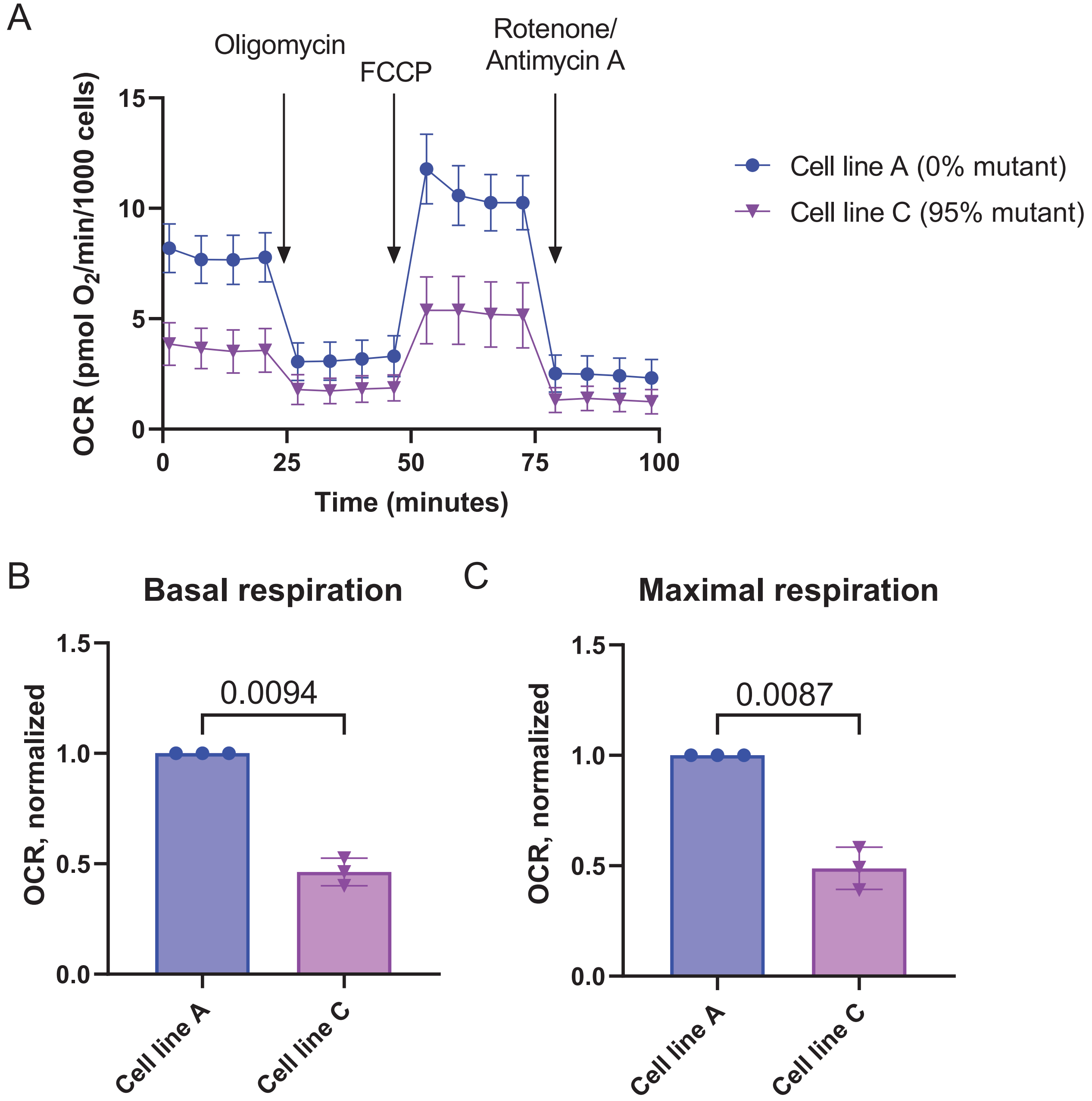

Supplemental Figure 2. 95% m.3243G mutant cybrid cells exhibit a respiratory impairment compared to wild-type cells.

(A) Seahorse Cell Mito Stress Test comparing cell lines A (0% mutant) and C (95% mutant). This assay measures oxygen consumption rates (OCR) of live cells at baseline and following the addition of various modulators of respiration. Oligomycin inhibits Complex V, leading to a drop in oxygen consumption corresponding to the oxygen required for mitochondrial ATP synthesis. FCCP dissipates the proton gradient and uncouples the activity of the mitochondrial respiratory chain from ATP synthesis, producing the cells' maximal respiratory capacity. Rotenone and Antimycin A inhibit complexes I and III, respectively, leading to a complete drop in mitochondrial-associated oxygen consumption. Timing of oligomycin, FCCP, and rotenone/Antimycin A additions are indicated with arrows.
(B) Basal respiration for cell line C, normalized to cell line A (WT). Basal respiration corresponds to timepoints #1-4, prior to Oligomycin injection.
(C) Maximal respiration values for cell line C, normalized to cell line A (WT). Maximal respiration corresponds to timepoints #9-12, immediately following FCCP injection.

Data encompass three independent experiments and are shown as mean ± SD. Statistical analysis was performed using a two-tailed t-test on the raw data.

WT, wild-type; OCR, oxygen consumption rate.

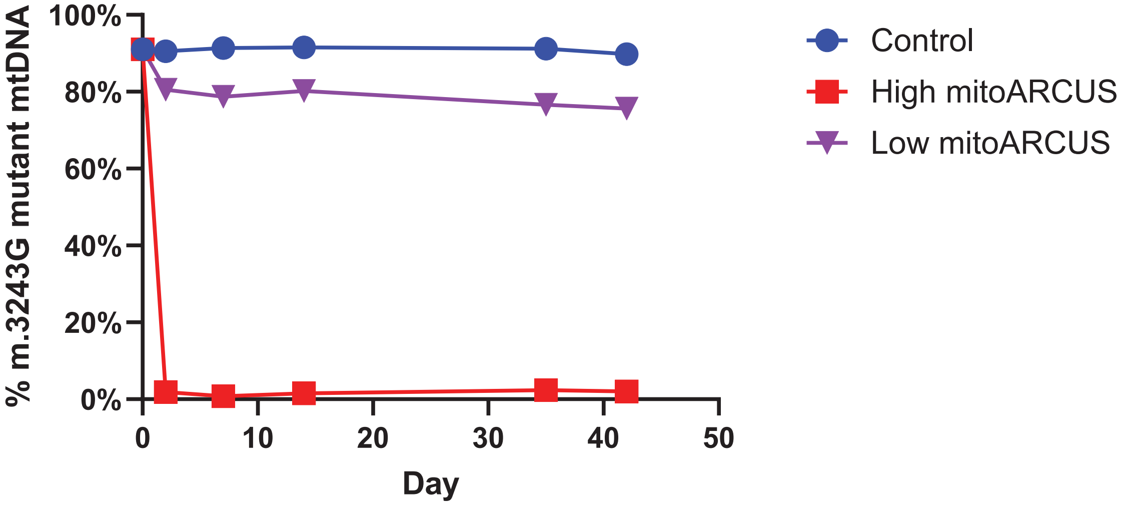

Supplemental Figure 3. Elimination of m.3243G mutant mtDNA is maintained over time.
A cybrid line harboring approximately 90% mutant mtDNA (m.3243G) was nucleofected with a low ($1x10^2$ mRNA copies/cell) or high ($1x10^4$ mRNA copies/cell) dose of mitoARCUS. mtDNA heteroplasmy was analyzed at different days in culture.

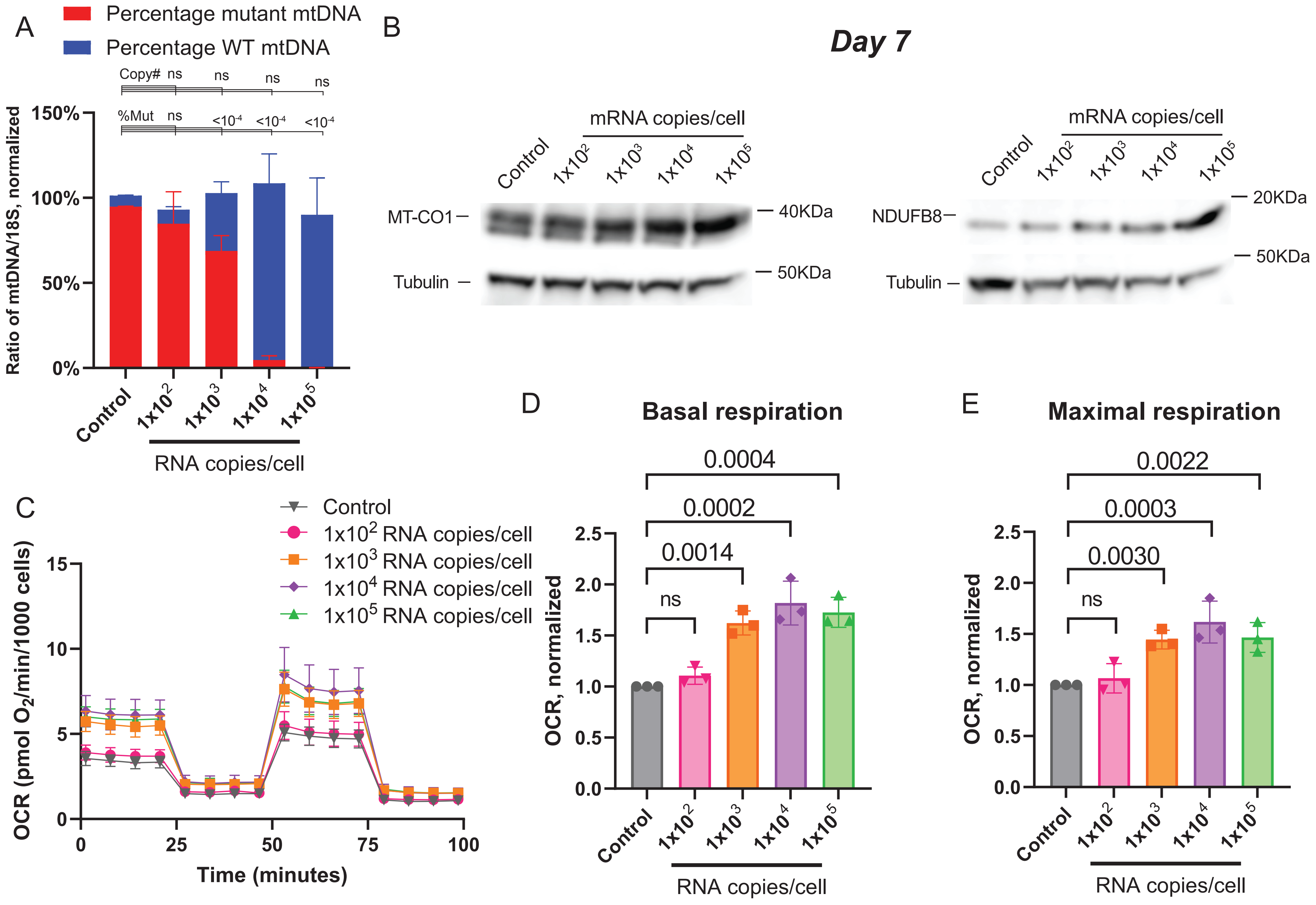

Supplemental figure 4. mitoARCUS3.2 shifts heteroplasmy in 95% m.3243G mutant cybrid cells, resulting in increased steady-state levels of mitochondrial proteins and increased respiration at day seven.

Cell line C (95% mutant) was nucleofected with either an MTS-GFP control or mitoARCUS3.2 at 10-fold mRNA dilutions, starting at 1x10□ mRNA copies/-cell. Cellular DNA was collected at day seven for mtDNA heteroplasmy and mtDNA copy number analysis (A), protein lysates were collected for Western blot (B), and live cells were analyzed for respiration (C-E).

(A) mtDNA heteroplasmy, normalized to mtDNA copy number of the control.
(B) Western blots showing MT-CO1, NDUFB8, and alpha-Tubulin steady-state levels.
(C) Seahorse Cell Mito Stress Test.
(D) Basal respiration, normalized to the control.
(E) Maximal respiration, normalized to the control.

Data encompass three independent experiments and are shown as mean ± SD. Statistical analysis was performed using an ANOVA on the raw data. Ns: P>0.05. All other P values indicated.

mtDNA, mitochondrial DNA; WT, wild-type; mut, mutant; OCR, oxygen consumption rate; 18S, 18S rDNA.

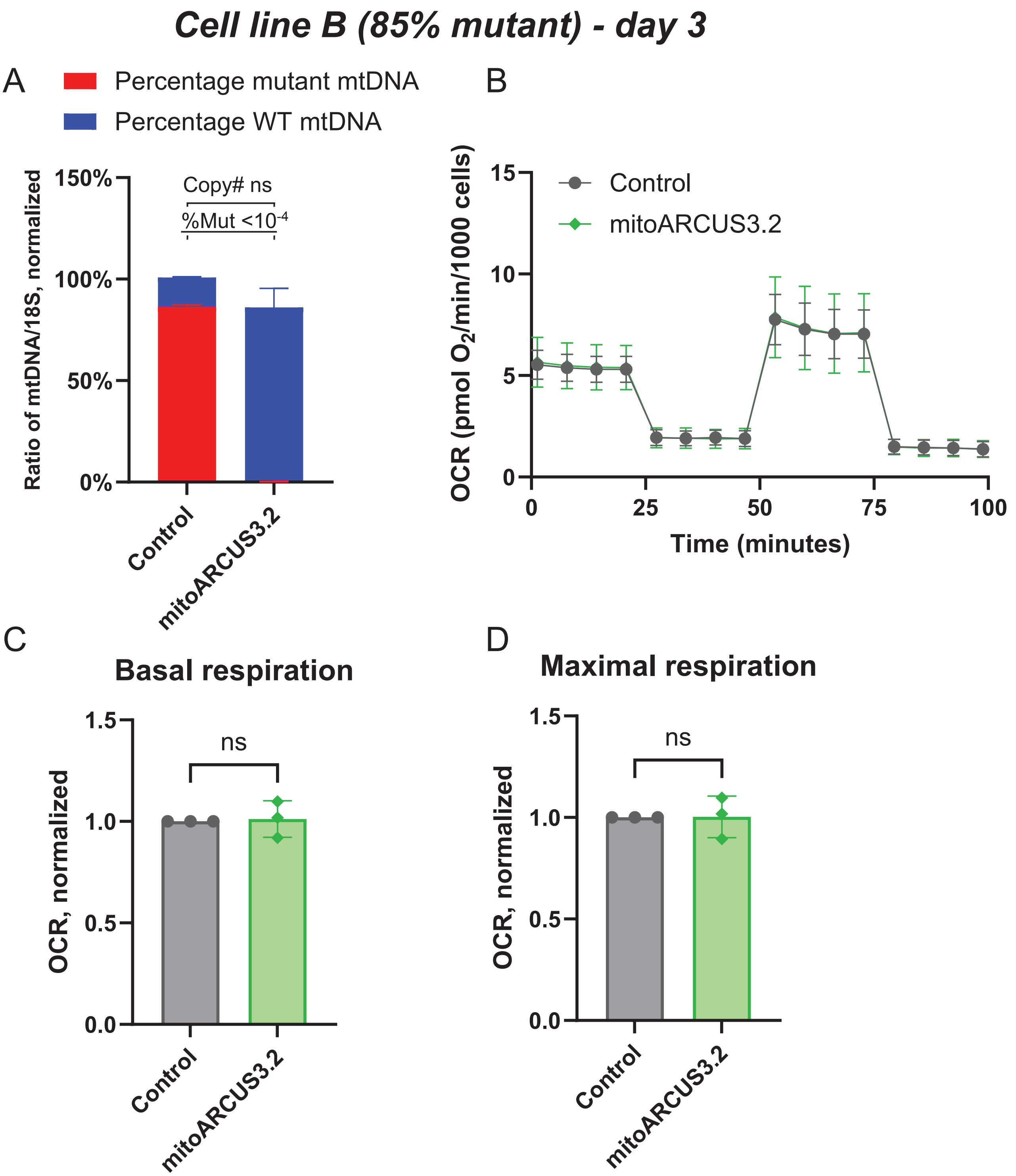

Supplemental Figure 5. mitoARCUS3.2 shifts heteroplasmy but does not improve respiration in 85% m.3243G mutant cybrid cells.
Cell line B (85% mutant) was nucleofected with mRNA encoding either MTS-GFP or mitoARCUS3.2 at a dose of 1x10□ mRNA copies/cell. Cellular DNA was collected at day three for mtDNA heteroplasmy and mtDNA copy number analysis (A) and live cells were analyzed for respiration (B-D).

(A) mtDNA heteroplasmy, normalized to mtDNA copy number of the control.
(B) Seahorse Cell Mito Stress Test.
(C) Basal respiration, normalized to the control.
(D) Maximal respiration, normalized to the control.

Data encompass three independent experiments and are shown as mean ± SD. Statistical analysis was performed using a two-tailed t-test on the raw data. Ns: $P>0.05$. All other P values indicated.

mtDNA, mitochondrial DNA; WT, wild-type; mut, mutant; OCR, oxygen consumption rate; 18S, 18S rDNA.

Cell line C (95% mutant) - day 3, galactose

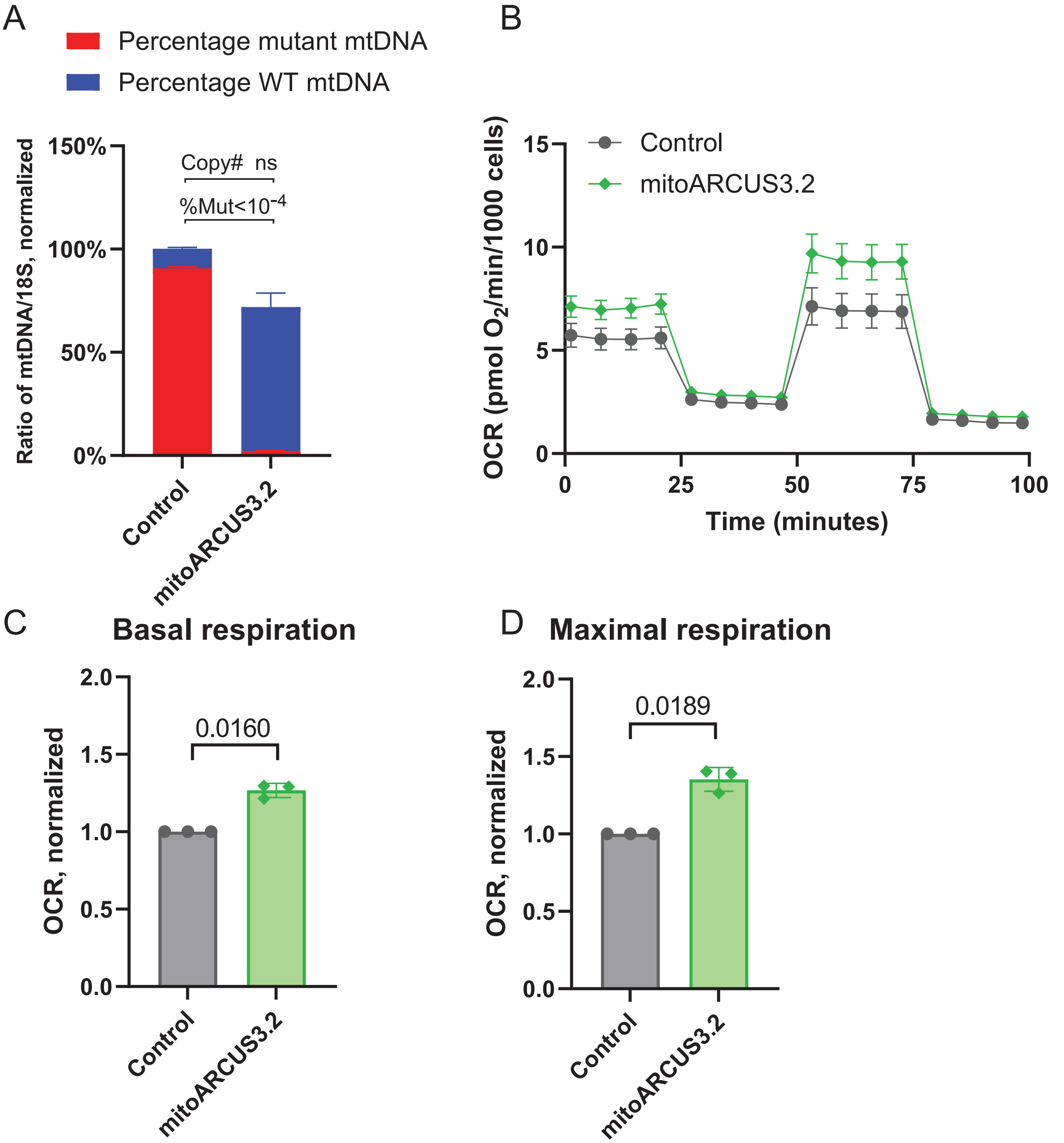

Supplemental Figure 6. 95% m.3243G mutant cybrid cells treated with mitoARCUS3.2 and grown in galactose exhibit improvements to respiration at day three.
Cell line C (95% mutant) was nucleofected with mRNA encoding either MTS-GFP or mitoARCUS3.2 at a dose of 1x10□ mRNA copies/cell. Cellular DNA was collected at day three for mtDNA heteroplasmy and mtDNA copy number analysis (A) and live cells were analyzed for respiration (B-D).

(A) mtDNA heteroplasmy, normalized to mtDNA copy number of the control.
(B) Seahorse Cell Mito Stress Test.
(C) Basal respiration, normalized to the control.
(D) Maximal respiration, normalized to the control.

Data encompass three independent experiments and are shown as mean ± SD. Statistical analysis was performed using a two-tailed t-test on the raw data. Ns: $P>0.05$. All other P values indicated.

mtDNA, mitochondrial DNA; WT, wild-type; OCR, oxygen consumption rate; 18S, 18S rDNA.

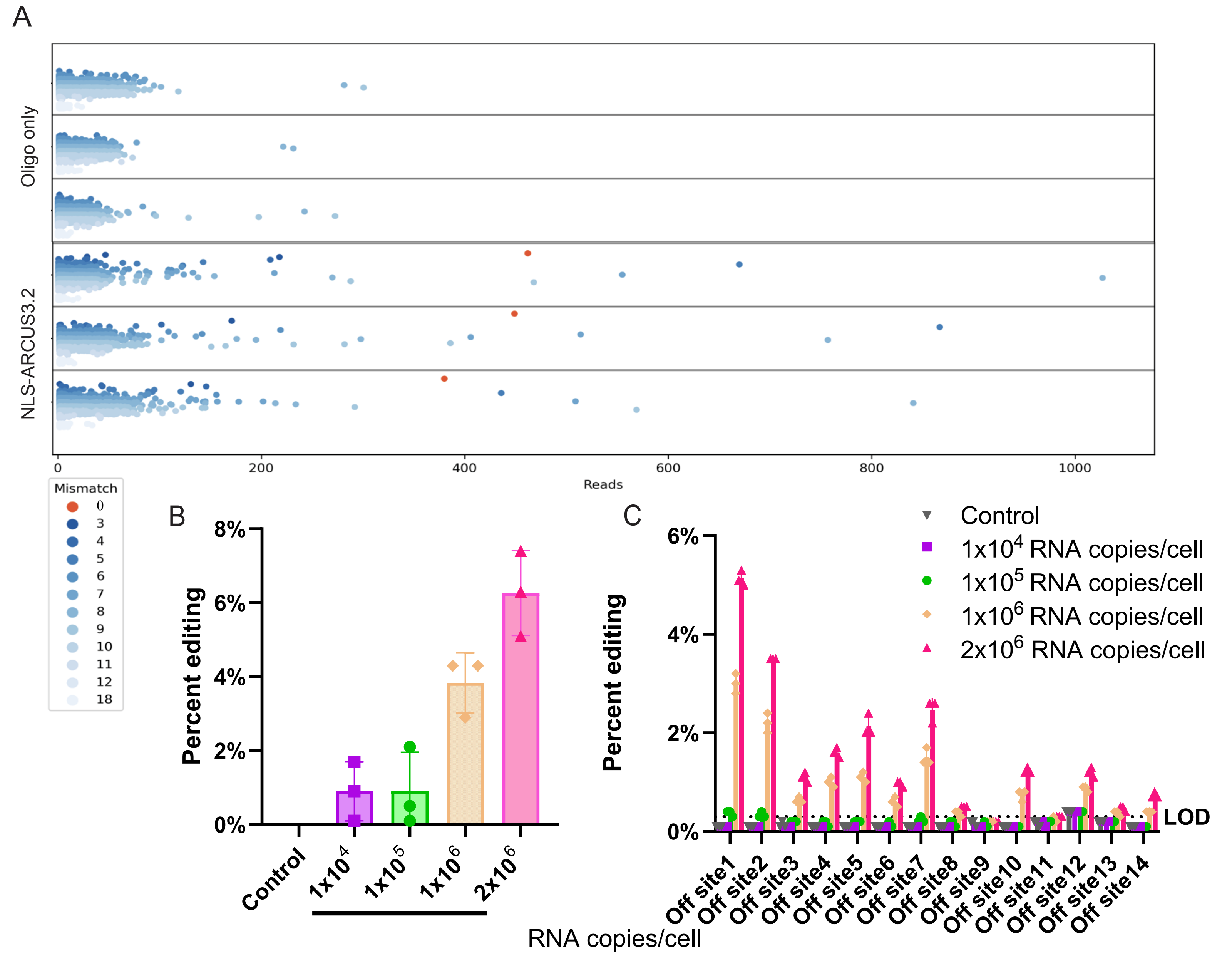

Supplemental Figure 7. Identification and characterization of potential nuclear off-target sequences cleaved by NLS-ARCUS3.2.
HEK 293 cells modified to contain the mitoARCUS binding site on the nuclear chromosome were used to identify and characterize potential nuclear off-target sites cleaved by a nuclear-localized ARCUS3.2.

(A) Oligo capture data for the control (oligo only) and NLS-ARCUS3.2 in modified HEK 293 cells. The red dot represents the introduced on-target (mutant m.3243G) sequence within the nuclear genome, while each blue dots represents a unique nuclear off-target site that incorporated an oligo. Darker blue dots have fewer mis-matches from the intended sequence, while lighter blue dots have more mismatches. The x-axis indicates the number of reads that were recovered for each site. NLS-ARCUS3.2 mRNA was transfected at a dose of 1x10[illegible] mRNA copies/cell along with a saturating dose of the dsDNA oligo pool.
(B) Indels generated at the introduced on-target site by NLS-ARCUS3.2 at various mRNA doses in modified HEK 293 cells, without co-transfection with the dsDNA oligo.
(C) Indels generated at each of the 14 identified off-target sites with NLS-ARCUS3.2 at various mRNA doses in modified HEK 293 cells, without co-transfection with the dsDNA oligo.

Data encompass three independent experiments and are shown as mean ± SD.

NLS, nuclear localization signal; indel, insertion and/or deletion; dsDNA, double-stranded DNA; LOD, limit of detection.

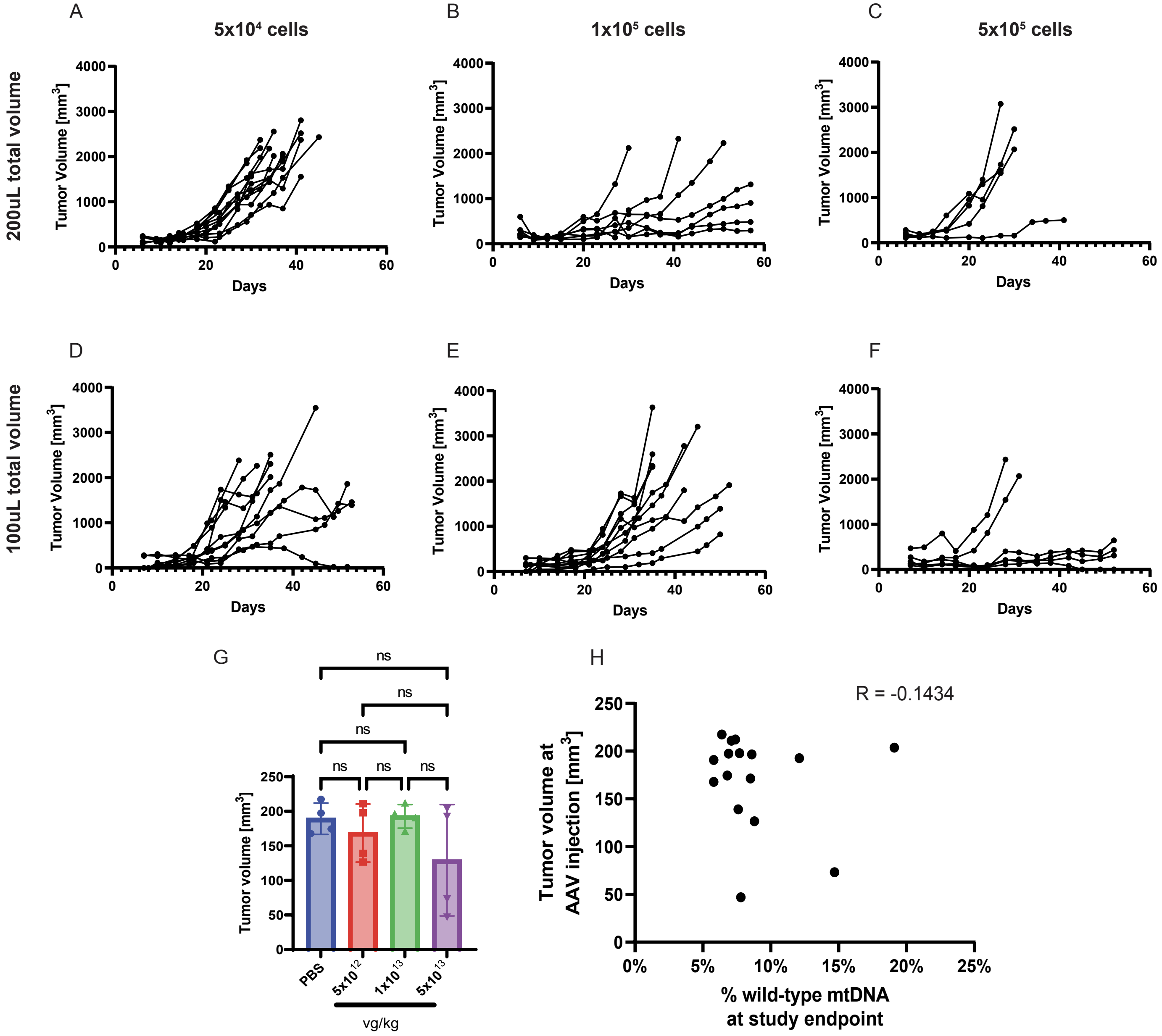

Supplemental Figure 8. Tumor volume at time of AAV injection does not impact heteroplasmy shift in xenograft mouse model.

Cell line C (95% mutant) was used to generate a xenograft in nude mice. Various cell doses and injection volumes were evaluated for consistent tumor generation. Once the model was established, AAV was used to deliver mitoARCUS3.2 systemically at various doses. Caliper measurements were used to evaluate growth of the tumors throughout the duration of the study.

(A) Tumor volume over time, generated by injection of $5x10^4$ cells in 200uL total volume.
(B) Tumor volume over time, generated by injection of $1x10^5$ cells in 200uL total volume.
(C) Tumor volume over time, generated by injection of $5x10^5$ cells in 200uL total volume.
(D) Tumor volume over time, generated by injection of $5x10^4$ cells in 100uL total volume.
(E) Tumor volume over time, generated by injection of $1x10^5$ cells in 100uL total volume.
(F) Tumor volume over time, generated by injection of $5x10^5$ cells in 100uL total volume.
(G) Tumor volume at the time of AAV injection (day 18); n=4.
(H) Relationship between the tumor volume at the time of AAV injection (day 18) and the level of wild-type mtDNA present in the tumor at study endpoint (day 35).

Data are shown as mean ± SD. Statistical analysis was performed using one way ANOVA, ns: P>0.05. Correlation was measured using Pearson's correlation coefficient (R).

mtDNA, mitochondrial DNA; AAV, adeno-associated virus.

Supplemental Table 1 – Nuclear off-target sites

Chromosomal and nucleotide position for each of the 14 evaluated nuclear off-target sites

**Off-target site**	**Chromosome**	**Nucleotide position**
Off site 1	4	169089489
Off site 2	7	113793556
Off site 3	1	237835920
Off site 4	15	44097475
Off site 5	8	93003183
Off site 6	3	101253660
Off site 7	11	64848681
Off site 8	8	23985031
Off site 9	5	123557482
Off site 10	5	68766410
Off site 11	5	91385034
Off site 12	9	19115132
Off site 13	12	6610981
Off site 14	12	18095338

Supplemental Table 2.

Oligonucleotides used in this study.

Quantification of mtDNA copy number and mtDNA linearization

Assay	Primer/probe	Sequence (5'-3')
mtDNA reference – MT-ND2	Probe	/5HEX/AGCAGTTCT/ZEN/ACCGTACAACCCTAACA/3IABkFQ/
	Forward primer	GGCAGTTGAGGTGGATTA
	Reverse primer	GGAATGCGGTAGTAGTTAGG
nDNA reference – 18S rDNA	Probe	/56-FAM/ AACCAGACA/ZEN/AATCGCTCCACCAAC/3IABkFQ/
	Forward primer	CGGACAGGATTGACAGATT
	Reverse primer	CCAGAGTCTCGTTCGTTATC
mitoARCUS binding site – MT-TL1	Probe (BHQPlus)	/FAM/TGGCAGGGCCCGGT/BHQplus/
	Forward primer	CCCAAGAACAGGGTTTGTTAAG
	Reverse primer	GGAATGCCATTGCGATTAG

Nuclear off-target quantification

Assay	Primer/probe	Sequence (5'-3')
Introduced nDNA binding site	Probe (BHQPlus)	/FAM/TGGCAGGGCCCGGT/BHQplus/
	Forward primer	AGAGCTCTCTGGCTAACTA
	Reverse primer	GGAATGCCATTGCGATTAG
Reference	Probe	/5HEX/AGCAGTTCT/ZEN/ACCGTACAACCCTAACA/3IABkFQ/
	Forward primer	GGCAGTTGAGGTGGATTA
	Reverse primer	GAATGACACCTACTCAGACAA

Quantification of AAV copy number

Assay	Primer/probe	Sequence (5'-3')
AAV DNA	Probe	/5HEX/TGGACAAAC/ZEN/CACAACTAGAATGCAGTG/3IABkFQ/
	Forward primer	CAGGATCCAGACATGATAAGA

	Reverse primer	GCAATAGCATCACAAATTTCAC
Mouse nDNA - TTR	Probe	/56-FAM/CCTCGCTGG/ZEN/ACTGGTATTTGTGTCT/3IABkFQ/
	Forward primer	GACAGGATGGCTTCCCTTC
	Reverse primer	GTCTAACTGCCATGTCTGGAT
Human nDNA – APOC3	Probe	/56-FAM/AGCCCCGGG/ZEN/TACTCCTTGTT/3IABkFQ/
	Forward primer	TTCCTTGCAGGAACAGAG
	Reverse primer	CTGCTTGACCACCCATT

Quantification of mouse mtDNA copy number

Assay	Primer/probe	Sequence (5’-3’)
mtDNA reference – MT-ND5	Probe	/5HEX/ACCCAATCA/ZEN/AACGCCTAGCATTCG/3IABkFQ/
	Forward primer	CCTGAGCCCTACTAATTACAC
	Reverse primer	GAGATGACAAATCCTGCAAAG
nDNA reference – 18S rDNA	Probe	/56-FAM/AGAAACGGC/ZEN/TACCACATCCAAGGA/3IABkFQ/
	Forward primer	CGTCTGCCCTATCAACTTT
	Reverse primer	CCTCGAAAGAGTCCTGTATTG
