# Supplementary material for: Efficient elimination of MELAS-associated m.3243G mutant mitochondrial DNA by an engineered mitoARCUS nuclease
Source: Nat Metab. 2023 Nov 30;5(12):2169–83. doi: 10.1038/s42255-023-00932-6 (PMC10730414; doi:10.1038/s42255-023-00932-6)
Supplement: Supplementary file 2 — Reporting Summary [file 42255_2023_932_MOESM2_ESM.pdf]

nature portfolio

Corresponding author(s): Cassandra L. Gorsuch, Carlos T. Moraes

Last updated by author(s): September 13, 2023

Reporting Summary

Nature Portfolio wishes to improve the reproducibility of the work that we publish. This form provides structure for consistency and transparency in reporting. For further information on Nature Portfolio policies, see our Editorial Policies and the Editorial Policy Checklist.

Statistics

For all statistical analyses, confirm that the following items are present in the figure legend, table legend, main text, or Methods section.

n/a	Confirmed	
☐	☒	The exact sample size (*n*) for each experimental group/condition, given as a discrete number and unit of measurement
☐	☒	A statement on whether measurements were taken from distinct samples or whether the same sample was measured repeatedly
☐	☒	The statistical test(s) used AND whether they are one- or two-sided<br>*Only common tests should be described solely by name; describe more complex techniques in the Methods section.*
☐	☒	A description of all covariates tested
☐	☒	A description of any assumptions or corrections, such as tests of normality and adjustment for multiple comparisons
☐	☒	A full description of the statistical parameters including central tendency (e.g. means) or other basic estimates (e.g. regression coefficient) AND variation (e.g. standard deviation) or associated estimates of uncertainty (e.g. confidence intervals)
☐	☒	For null hypothesis testing, the test statistic (e.g. *F*, *t*, *r*) with confidence intervals, effect sizes, degrees of freedom and *P* value noted<br>*Give P values as exact values whenever suitable.*
☒	☐	For Bayesian analysis, information on the choice of priors and Markov chain Monte Carlo settings
☒	☐	For hierarchical and complex designs, identification of the appropriate level for tests and full reporting of outcomes
☐	☒	Estimates of effect sizes (e.g. Cohen's *d*, Pearson's *r*), indicating how they were calculated

Our web collection on statistics for biologists contains articles on many of the points above.

Software and code

Policy information about availability of computer code

Data collection	BioRad digital PCR Quantasoft 1.0596; SeaHorse Wave 2.6.0
Data analysis	GraphPad Prism; NGS Nuclear off-target analysis by oligo capture- NGS reads were bwa-mem(http://bio-bwa.sourceforge.net/bwa. shtml) and further analyzed as described in ref.47.; BioRad digital PCR Quantasoft 1.0596

For manuscripts utilizing custom algorithms or software that are central to the research but not yet described in published literature, software must be made available to editors and reviewers. We strongly encourage code deposition in a community repository (e.g. GitHub). See the Nature Portfolio guidelines for submitting code & software for further information.

Data

Policy information about availability of data

All manuscripts must include a data availability statement. This statement should provide the following information, where applicable:

- Accession codes, unique identifiers, or web links for publicly available datasets
- A description of any restrictions on data availability
- For clinical datasets or third party data, please ensure that the statement adheres to our policy

Data available upon request with appropriate agreements.

Research involving human participants, their data, or biological material

Policy information about studies with human participants or human data. See also policy information about sex, gender (identity/presentation), and sexual orientation and race, ethnicity and racism.

Reporting on sex and gender	N.A.
Reporting on race, ethnicity, or other socially relevant groupings	N.A.
Population characteristics	N.A.
Recruitment	N.A.
Ethics oversight	N.A.

Note that full information on the approval of the study protocol must also be provided in the manuscript.

Field-specific reporting

Please select the one below that is the best fit for your research. If you are not sure, read the appropriate sections before making your selection.

☒ Life sciences ☐ Behavioural & social sciences ☐ Ecological, evolutionary & environmental sciences

For a reference copy of the document with all sections, see nature.com/documents/nr-reporting-summary-flat.pdf

Life sciences study design

All studies must disclose on these points even when the disclosure is negative.

Sample size	Sample size based on historic results on similar analysis (e.g. refs. 39, 67).
Data exclusions	No data exclusion
Replication	Key experiments were repeated multiple times (at least 3 times). No replications excluded as the results were reproducible.
Randomization	Animals and samples were assigned randomly to experimental groups.
Blinding	Not blinded. Measurements were objective.

Reporting for specific materials, systems and methods

We require information from authors about some types of materials, experimental systems and methods used in many studies. Here, indicate whether each material, system or method listed is relevant to your study. If you are not sure if a list item applies to your research, read the appropriate section before selecting a response.

Materials & experimental systems

n/a	Involved in the study	
☐	☒	Antibodies
☐	☒	Eukaryotic cell lines
☒	☐	Palaeontology and archaeology
☐	☒	Animals and other organisms
☒	☐	Clinical data
☒	☐	Dual use research of concern
☒	☐	Plants

Methods

n/a	Involved in the study	
☒	☐	ChIP-seq
☒	☐	Flow cytometry
☒	☐	MRI-based neuroimaging

Antibodies

Antibodies used	Mouse monoclonal anti-MT-CO1 [1D6E1A8] (ab14705 Abcam, Cambridge, UK, 1:1,000 dilution), mouse monoclonal anti-NDUFB8 [20E9DH10C12] (ab110242 Abcam, 1:1,000 dilution), and mouse monoclonal anti-alpha Tubulin [DM1A] (ab7291 Abcam, 1:20,000 dilution). The secondary antibody was goat anti-mouse IgG H&L (HRP) (ab205719 Abcam, 1:5,000 dilution).

Validation	All antibodies used have been characterized in previous publications (refs 36, 40) and tested in cells lacking mtDNA (DOI: https://doi.org/10.1074/jbc.M805972200).

Eukaryotic cell lines

Policy information about cell lines and Sex and Gender in Research

Cell line source(s)	Osteosarcoma cybrids (143B nuclear background). mtDNA was introduced as described in previous publications (Mol Cell Biol. 1992 :480-90. Defects in mitochondrial protein synthesis and respiratory chain activity segregate with the tRNA(Leu(UUR)) mutation associated with mitochondrial myopathy, encephalopathy, lactic acidosis, and strokelike episodes. M P King , Y Koga, M Davidson, E A Schon; Biochim Biophys Acta. 1992 Jul 17;1101(2):206-9. The mitochondrial tRNA(Leu)(UUR)) mutation in MELAS: a model for pathogenesis. E A Schon, Y Koga, M Davidson, C T Moraes, M P King.<br><br>FLP-IN 293 (ThermoFisher-Invitrogen).
Authentication	Were authenticated cybrids for the mtDNA genotype. PCR primers specific for the mtDNA encompassing a unique SNV, which was then identified by RFLP and digital PCR. We autheticated the Flp-In 293 by detecting the insert correctly incorporated in the target site (done by PCR).
Mycoplasma contamination	Regularly tested. Negative for mycoplasma.
Commonly misidentified lines<br>(See ICLAC register)	Flp-In™ 293 was purchased from Thermo and used to insert a DNA in the Flp flanked site. As the DNA was correctly inserted, as detected by PCR, we assumed the cell identity was correct.

Animals and other research organisms

Policy information about studies involving animals; ARRIVE guidelines recommended for reporting animal research, and Sex and Gender in Research

Laboratory animals	Mus musculus (J:NU- 007850-JAX) 9-11 weeks old. Temp and Humidity added to manuscript.
Wild animals	No wild animals were used in the study.
Reporting on sex	Only females used for homogeneity. Mice used as tumor hosts only.
Field-collected samples	No field collected samples were used in the study.
Ethics oversight	Approved by the I.A.C.U.C.- Mispro Biotech

Note that full information on the approval of the study protocol must also be provided in the manuscript.
