# Supplementary material for: Efficient elimination of MELAS-associated m.3243G mutant mitochondrial DNA by an engineered mitoARCUS nuclease
Source: Nat Metab. 2023 Nov 30;5(12):2169–83. doi: 10.1038/s42255-023-00932-6 (PMC10730414; doi:10.1038/s42255-023-00932-6)

MTCO1(MTCO1 blot) day 3 and 7
(Figure 3 and Supplemental Figure S4)

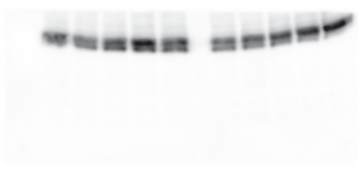

Tubulin (MTCO1 blot) day 3 and 7
(Figure 3 and Supplemental Figure S4)

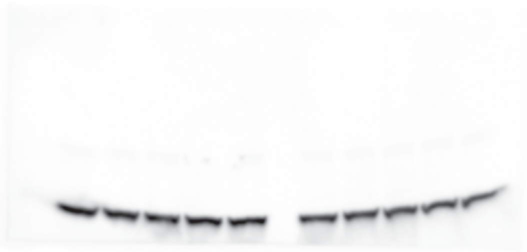

Ndufb8 day 3
(Figure 3)

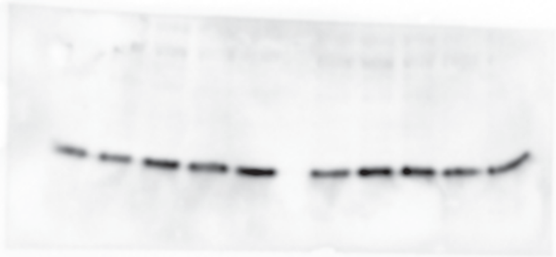

Tubulin day 3
(Figure 3)

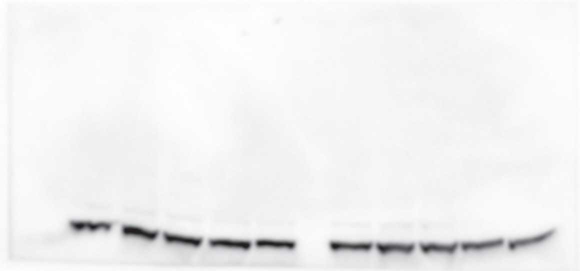

Ndufb8 day 7
(Figure S4)

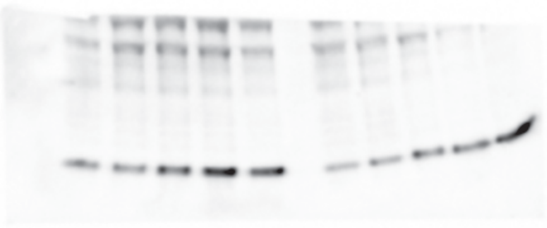

Tubulin day 7
(Figure S3)

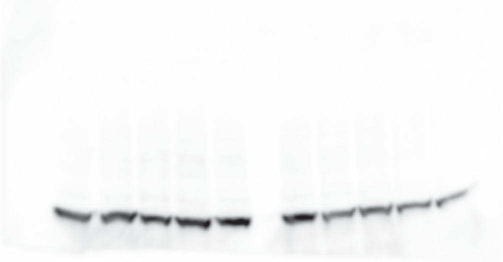

Supplement: Supplementary file 4 — Unprocessed immunoblots. [file 42255_2023_932_MOESM4_ESM.pdf]
